# Apt interpretation of comprehensive lipoprotein data in large-scale epidemiology: disclosure of fundamental structural and metabolic relationships

**DOI:** 10.1093/ije/dyab156

**Published:** 2021-08-18

**Authors:** Mika Ala-Korpela, Siyu Zhao, Marjo-Riitta Järvelin, Ville-Petteri Mäkinen, Pauli Ohukainen

**Affiliations:** Computational Medicine, Faculty of Medicine, University of Oulu, Oulu, Finland; Center for Life Course Health Research, Faculty of Medicine, University of Oulu, Oulu, Finland; Biocenter Oulu, University of Oulu, Oulu, Finland; NMR Metabolomics Laboratory, School of Pharmacy, University of Eastern Finland, Kuopio, Finland; Computational Medicine, Faculty of Medicine, University of Oulu, Oulu, Finland; Center for Life Course Health Research, Faculty of Medicine, University of Oulu, Oulu, Finland; Biocenter Oulu, University of Oulu, Oulu, Finland; Center for Life Course Health Research, Faculty of Medicine, University of Oulu, Oulu, Finland; Biocenter Oulu, University of Oulu, Oulu, Finland; Unit of Primary Health Care, Oulu University Hospital, OYS, Oulu, Finland; Department of Epidemiology and Biostatistics, MRC-PHE Centre for Environment and Health, School of Public Health, Imperial College London, London, UK; Department of Life Sciences, College of Health and Life Sciences, Brunel University London, UK; Australian Centre for Precision Health, University of South Australia, Adelaide, Australia; Computational and Systems Biology Program, Precision Medicine Theme, South Australian Health and Medical Research Institute, Adelaide, Australia; Computational Medicine, Faculty of Medicine, University of Oulu, Oulu, Finland; Center for Life Course Health Research, Faculty of Medicine, University of Oulu, Oulu, Finland; Biocenter Oulu, University of Oulu, Oulu, Finland

**Keywords:** NMR spectroscopy, lipoprotein subclasses, lipoprotein lipids, lipoprotein composition, apolipoproteins, metabolomics

## Abstract

**Background:**

Quantitative lipoprotein analytics using nuclear magnetic resonance (NMR) spectroscopy is currently commonplace in large-scale studies. One methodology has become widespread and is currently being utilized also in large biobanks. It allows the comprehensive characterization of 14 lipoprotein subclasses, clinical lipids, apolipoprotein A-I and B. The details of these data are conceptualized here in relation to lipoprotein metabolism with particular attention on the fundamental characteristics of subclass particle numbers, lipid concentrations and compositional measures.

**Methods and Results:**

The NMR methodology was applied to fasting serum samples from Northern Finland Birth Cohorts 1966 and 1986 with 5651 and 5605 participants, respectively. All results were highly consistent between the cohorts. Circulating lipid concentrations in a particular lipoprotein subclass arise predominantly as the result of the circulating number of those subclass particles. The spherical lipoprotein particle shape, with a radially oriented surface monolayer, imposes size-dependent biophysical constraints for the lipid composition of individual subclass particles and inherently restricts the accommodation of metabolic changes via compositional modifications. The new finding that the relationship between lipoprotein subclass particle concentrations and the particle size is log-linear reveals that circulating lipoprotein particles are also under rather strict metabolic constraints for both their absolute and relative concentrations.

**Conclusions:**

The fundamental structural and metabolic relationships between lipoprotein subclasses elucidated in this study empower detailed interpretation of lipoprotein metabolism. Understanding the intricate details of these extensive data is important for the precise interpretation of novel therapeutic opportunities and for fully utilizing the potential of forthcoming analyses of genetic and metabolic data in large biobanks.

Key Messages

- The circulating particle number of a lipoprotein subclass is the defining measure for its lipid concentrations; the particle lipid composition plays only a minor role. The relationship between circulating lipoprotein subclass particle concentrations and the particle size is log-linear.
- The overall structure of lipoprotein subclass particles with a spherical shape and an oriented surface monolayer poses strong size-dependent *biomolecular constraints* for their lipid composition.
- The circulating lipoprotein subclass particle concentrations in humans are *metabolically constrained* for both elemental absolute and relative concentration ranges.
- The smallest high-density lipoprotein (HDL) particle concentrations are negatively associated with those of large HDL particles and generally the associations of the smallest HDL particles are similar to those of apolipoprotein B-containing particles.
- The apolipoprotein B-containing particles constitute <10% of all lipoprotein particles but carry around two-thirds of the circulating lipoprotein lipids. Low-density lipoprotein and intermediate-density lipoprotein particles amount to almost 90% of all apolipoprotein B-containing particles.
- The supplemental role of lipoprotein subclass data in cardiometabolic risk assessment is slight.
- In the current era of biobanks and big data, the combination of lipoprotein subclass data with drug-target Mendelian randomization analyses provides great scientific synergy, intricate details and potential cost savings in drug development.

## Introduction

The role of cholesterol in the development of human atherosclerosis has been known for >100 years. After the original findings in relation to cholesterol, it took almost 50 years to link cholesterol to circulating low-density lipoprotein (LDL) particles and reveal the association of LDL cholesterol with coronary heart disease (CHD) in the 1950s. LDL cholesterol and a few other lipoprotein measures, particularly triglycerides and high-density lipoprotein (HDL) cholesterol, have been in the spotlight of CHD and related pharmacological research since then.^1^

Broader research on the molecular characteristics of circulating lipoprotein particles began after the invention of using sequential preparative ultracentrifugation to physically isolate native lipoprotein particles according to their densities and then further characterizing them chemically.^2^ The micellar structure of lipoprotein particles was suggested in 1968 on the basis of nuclear magnetic resonance (NMR) spectroscopy experiments.^3^ This eventually led to the perception of lipoproteins as spherical particles of a non-polar lipid filled core and a surface with radially oriented lipids, together with various apolipoprotein molecules, with polar molecular groups allowing them to be dissolved in a water-based environment, i.e. blood plasma.^4^^,^^5^

Although ultracentrifugation became the workhorse in lipoprotein analytics, the tediousness of the process, particularly with a tight density scale, prevented large studies with a high resolution of lipoprotein fractions.^4^ However, in the 1990s, NMR spectroscopy was introduced as a cost-effective methodology for acquiring detailed large-scale data on lipoprotein subclasses. A few methodologies were developed with slightly different definitions and numbers of lipoprotein subclasses.^6^^,^^7^ The methodology invented and developed in our research team^6^ was extended to a metabolomics platform to quantify >200 metabolic measures, including comprehensive characterization of 14 lipoprotein subclasses. Over the last 10 years, this particular platform has become common in large-scale epidemiology and genetics,^8–10^ and it is currently being applied to analysing all 500 000 serum samples in the UK Biobank, with >100 000 samples already analysed by Nightingale Health Ltd.^11^ Some 300 publications with applications of this methodology are currently available. When the extensive and unique lipoprotein data become publicly available in the UK Biobank, the number of publications is likely to surge.

Almost all of the epidemiological and genetic applications of the lipoprotein data have focused on concentration measures only, even though the compositional data are automatically incorporated into the analytical results for each serum sample.^9^^,^^10^ One reason for the reluctance of researchers to include the compositional data may be their unfamiliarity both in epidemiology and clinical chemistry, as well as clinical studies. In addition, the concentration data for 14 lipoprotein subclasses already consist of 98 measures (plus apolipoproteins B and A-I as well as various standard lipoprotein lipid measures); thus, adding 70 compositional measures considerably complicates statistical analyses and the presentation of results. Nevertheless, recent applications have indicated that a detailed understanding of the treatment effects of novel lipid-modifying drug targets would benefit remarkably from high-resolution comprehensive molecular data on lipoprotein subclasses. The compositional data may have an independent role in the risk assessment and understanding of lipoprotein metabolisms.^12–15^ It is therefore pertinent to discuss the comprehensive lipoprotein subclass data of this particular NMR platform in detail and to specify the fundamental characteristics of the concentration and compositional data and their relationships. We do this for the first time in this work.

## Methods

### Lipoprotein subclasses by NMR spectroscopy

The separation of lipoprotein subclasses by proton NMR spectroscopy is based on particle size. In more detail, the anisotropy of the magnetic susceptibility, due to the radially oriented surface monolayer of the spherical particles, inherently leads to a size-dependent frequency shift for the NMR resonances of lipoprotein lipids.^16^ The NMR data in this particular platform are calibrated via high-performance liquid chromatography with the platform resolution being 14 lipoprotein subclasses, defined by their particle size as follows: six very-low-density lipoprotein (VLDL) particle categories [extremely large (XXL-VLDL, with average particle diameter >75 nm), very large (XL-VLDL, 64 nm), large (L-VLDL, 53.6 nm), medium (M-VLDL, 44.5 nm), small (S-VLDL, 36.8 nm) and very small (XS-VLDL, 31.3 nm)]; intermediate-density lipoprotein particles (IDL, 28.6 nm), three LDL particle categories [large (L-LDL, 25.5 nm), medium (M-LDL, 23.0 nm) and small (S-LDL, 18.7 nm)]; and four HDL particle categories [very large (XL-HDL, 14.3 nm), large (L-HDL, 12.1 nm), medium (M-HDL, 10.9 nm) and small (S-HDL, 8.7 nm)].^9^^,^^17^ Independent verification for the robust NMR resolution with respect to the number of lipoprotein subclasses was published by Mihaleva and co-workers with an in-depth handling of the statistical grounds.^18^ Particle, triglyceride, phospholipid, cholesteryl ester and free cholesterol concentrations are quantified for all 14 subclasses, also allowing calculation of the relative lipid contents (i.e. lipid composition) for each lipoprotein subclass. The platform also provides all key traditional lipid measures (e.g. total triglycerides, total cholesterol and HDL cholesterol) as well as apolipoprotein B and A-I concentrations. This NMR metabolomics platform has been widely used in epidemiology and genetic studies over the last 10 years^9–11^^,^^17^ and the general methodological issues have been published and discussed previously.^8^^,^^17^^,^^19^ Analyses via this platform are commercially available from Nightingale Health Ltd.^11^

### Population cohorts

The Northern Finland Birth Cohort 1966 (NFBC66) with data for 5651 participants (at 46 years of age) were studied as the primary cohort and the NFBC86 with data for 5605 participants (at 16 years of age) as a replication cohort. More details on the NFBCs can be found in the Supplementary Note, including Supplementary Table S1 (available as Supplementary data at *IJE* online) for clinical characteristics. The selection or the traits of the cohorts are not important in this study focusing on overall lipoprotein subclass particle characteristics and associations as the existing literature already affirms them to be highly consistent in and between various large-scale studies.^9^^,^^10^^,^^12^^,^^13^^,^^17^^,^^20–24^

### Statistical analyses and concepts

The lipoprotein subclass information can be partitioned into three key categories of variables: (i) *circulating particle concentrations* (the particle numbers are within the range of 0.1 nmol/L and 10 µmol/L), (ii) *circulating lipid concentrations* (within the range of 0.1 and 10 mmol/L) and (iii) *particle lipid compositions* as the percentage of a certain lipid class of total lipids (mol%) per subclass particle. Apolipoprotein B is the key protein component in VLDL, IDL and LDL particles, and they are often called apolipoprotein B-containing particles. HDL particles do not contain apolipoprotein B but are abundant in apolipoprotein A-I. All lipoprotein particles also contain other apolipoprotein molecules, but they cannot be quantified by NMR.^25^ Spearman’s rank correlations (adjusted for sex) were used in all correlation analyses in this work. Twenty-four principal components explained >99% of the variation in the 174 lipoprotein measures (98 concentrations and 70 compositions for the 14 lipoprotein subclasses, 4 traditional lipid measures and apolipoprotein A-I and B). A multiple comparison Bonferroni-corrected *P*-value threshold of 0.002 (i.e. 0.05/24) was thus used to denote evidence in favour of an association.^26^ All statistical analyses were performed in the R statistical platform (version 3.6.2).

## Results

### Characteristics of lipoprotein subclass particles and their concentration attributes

**Figure 1:**
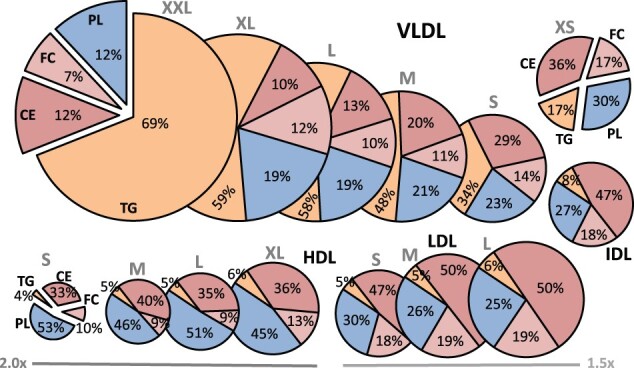
The relative lipid compositions of the 14 lipoprotein subclasses. The NMR platform resolution is 14 lipoprotein subclasses, defined by their particle size as follows: six VLDL particle categories; XXL-VLDL (with average particle diameter >75 nm), XL-VLDL (64 nm), L-VLDL (53.6 nm), M-VLDL (44.5 nm), S-VLDL (36.8 nm) and XS-VLDL (31.3 nm); IDL (28.6 nm), L-LDL (25.5 nm), M-LDL (23.0 nm) and S-LDL (18.7 nm); and XL-HDL (14.3 nm), L-HDL (12.1 nm), M-HDL (10.9 nm) and S-HDL (8.7 nm).^9^^,^^17^^,^^19^ Note that the size of the LDL and HDL particles in the figure is multiplied by 1.5 and 2.0, respectively. All the 14 lipoprotein subclass particles are drawn using the same size scale and the diameters highlighted in Figure 8. The data are mean values for 5651 participants in NFBC66. VLDL, very-low-density lipoprotein; IDL, intermediate-density lipoprotein; LDL, low-density lipoprotein; HDL, high-density lipoprotein; XXL, extremely large; XL, very large; L, large; M, medium; S, small; XS, very small; TG, triglycerides; PL, phospholipids; CE, cholesteryl esters; FC, free cholesterol.

**Figure 2:**
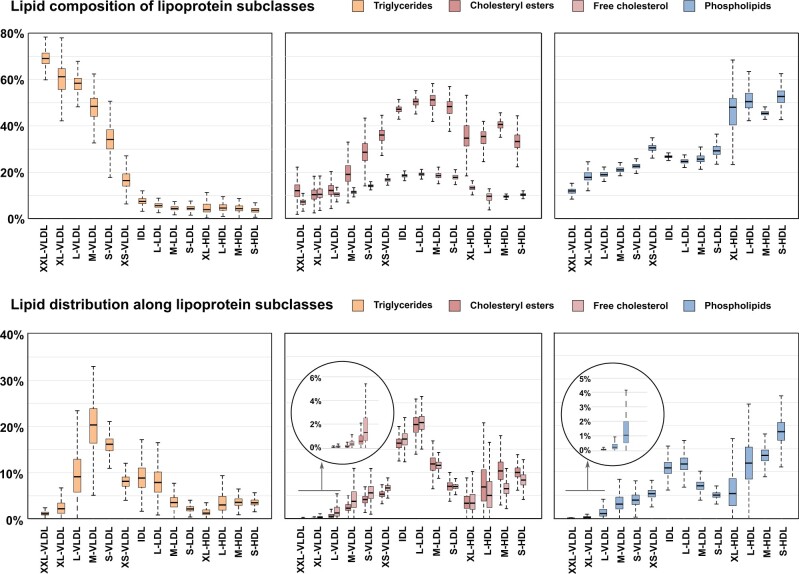
The compositional and circulatory characteristics of lipoprotein subclasses. The composition of lipoprotein subclasses depicts the relative lipid contents in each subclass particle category (i.e. the sum of the percentages of all the lipid classes for each subclass is 100%). The lipid distribution along the lipoprotein subclasses illustrates how a certain lipid class is distributed in the bloodstream among all the lipoprotein subclasses (i.e. the sum of all percentages in each inset is 100%). The data are from the NFBC66 including 5651 participants; each box plot shows the median within the interquartile range (IQR) and the minimum (Q1 – 1.5*IQR) and maximum (Q3 + 1.5*IQR) values with potential outliers. The abbreviations are as explained in the caption for Figure 1.

Figure 1 illustrates the 14 lipoprotein subclasses and their relative lipid content (see Supplementary Figure S1, available as Supplementary data at *IJE* online, for replication in the NFBC86). The compositional data are also visualized in the upper part of Figure 2 in comparison to the concentration data (identical data are shown for the NFBC86 in Supplementary Figure S2, available as Supplementary data at *IJE* online). The VLDL subclasses are the most triglyceride-enriched with variation from 69% to 17% of total lipids from XXL-VLDL to XS-VLDL. The IDL and LDL particles are all rich in cholesteryl esters (∼50%) as well as free cholesterol (∼20%). All HDL particles are rich in phospholipids (∼50%) and very poor in triglycerides.

Over 50% of circulating triglycerides are transported in VLDL particles, some 20% in IDL and LDL, and <20% in HDL particles (lower part of Figure 2 and Supplementary Figure S2, available as Supplementary data at *IJE* online). Roughly 50% of circulating cholesterol (both free and esterified) travels in IDL and LDL particles and some 20–30% in HDL particles. Around 50% of lipoprotein phospholipids in the circulation are in HDL particles. The size-dependent opposite compositional behaviour of triglycerides and cholesteryl esters in the apolipoprotein B-containing subclass particles is pronounced.

**Figure 3:**
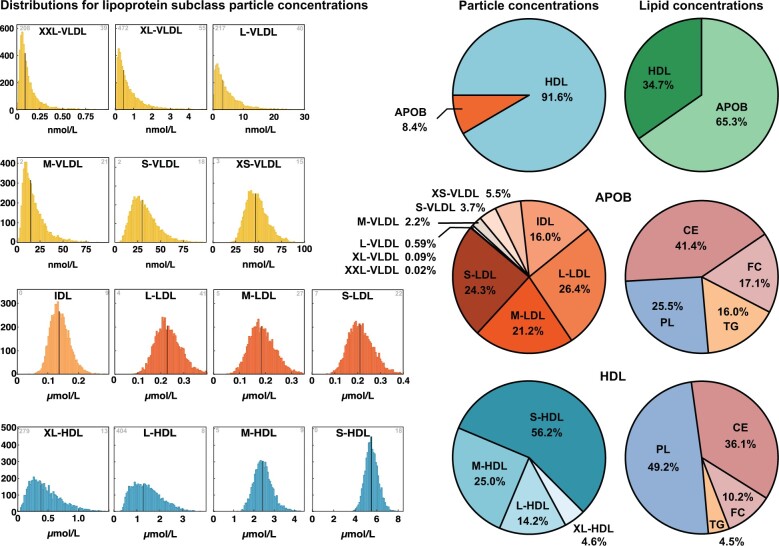
The distributions of particle concentrations for each lipoprotein subclass. The data are from the NFBC66 including 5651 participants. The grey number in the upper-left corner identifies the number of samples for which the particle concentration is zero and in the upper-right corner for how many high concentration values were cut off from the drawn distribution. Note that the concentration scale is nmol for the VLDL particles and µmol for the IDL, LDL and HDL particles. The black vertical lines denote the median concentration values. Various proportions of lipoprotein particles and lipids are shown in the pie charts as mean values for the 5651 participants in NFBC66. The abbreviations are as explained in the caption for Figure 1.

**Figure 4:**
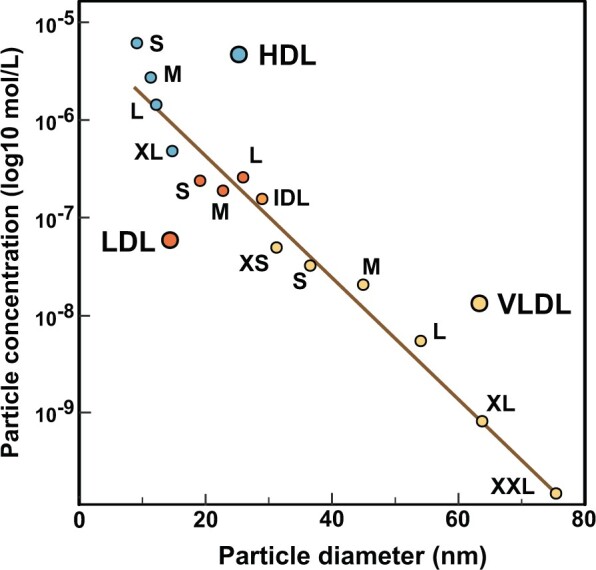
The log-linear relationship between the circulating lipoprotein subclass particle concentration and the particle diameter. The data and abbreviations are as explained in the caption for Figure 1.

The circulating subclass lipid concentrations depend on both the lipid composition of a subclass particle and the concentration (i.e. the number) of those particles in the bloodstream. The distributions for subclass particle concentrations are shown in Figure 3 (and in Supplementary Figure S3, available as Supplementary data at *IJE* online, for the NFBC86). The HDL particles are by far the most abundant with concentrations in a micromolar range, the IDL and LDL particles being at the range of 0.1–0.3 micromolar and the largest VLDL particles at the range of 0.1–10 nanomolar. Thus, on average, there are some 60 000 S-HDL and 1500 IDL particles per one XXL-VLDL particle in the circulation; 92% of all lipoprotein particles are HDL particles (Figure 3). Therefore, although the volume of an XXL-VLDL particle is ∼640 times that of an S-HDL particle (and 18 times that of an M-LDL particle), the variation in the particle lipid compositions within a subclass is clearly minor with respect to the variation in the corresponding particle concentrations. The circulating particle concentrations are therefore the defining measures for lipoprotein subclass lipid concentrations. These particle concentrations are log-linearly associated with the subclass particle size as illustrated in Figure 4. This intriguing finding denotes that the numbers of circulating lipoprotein particles are fundamentally restricted, both in a relative manner and in absolute concentrations (e.g. the number of VLDL particles must be appropriate for the circulating LDL cholesterol to remain within physiologically relevant limits).

**Figure 5:**
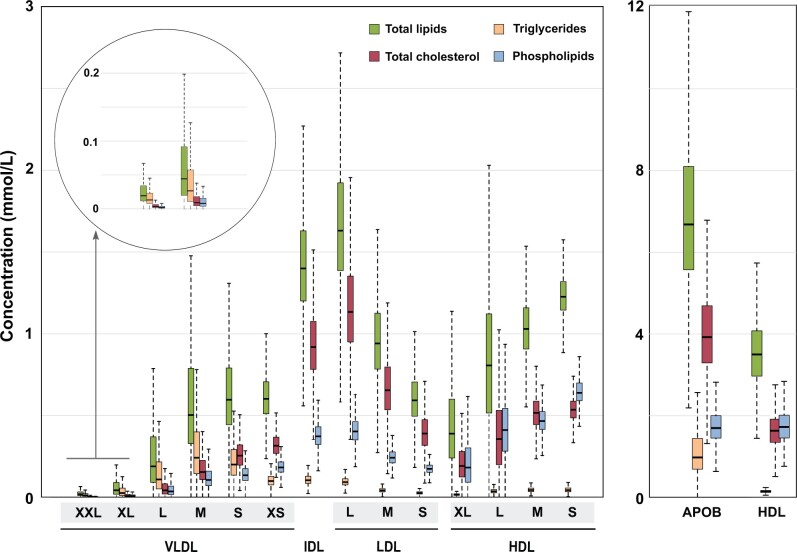
The absolute circulating lipid concentrations for each lipoprotein subclass and the corresponding summary measures for the apolipoprotein B-containing lipoprotein particles and HDL particles. The data and abbreviations are as explained in the captions for Figures 1 and 2.

An important distinction must be made between the concentration, i.e. number, of circulating particles (‘the particle view’) and the number of lipids that are transported within these particles (‘the cargo view’). This is due to large differences in the subclass particle transport volumes. As illustrated in Figure 3, ∼65% of all lipoprotein lipid cargo in the circulation is carried within the voluminous apolipoprotein B-containing particles though they comprise only <10% of the total particle number. HDL particles are markedly smaller than those with apolipoprotein B and are far more abundant in the circulation, accounting for >90% of all particles. On average, LDL particles amount to >70% and the three largest VLDL subclasses only less than a percent of circulating apolipoprotein B-containing particles. The smallest HDL particles constitute >50% of all circulating HDL particles. The apolipoprotein B-containing particles are most abundant in cholesteryl esters (>40 mol% of all lipids) and the HDL particles in phospholipids (almost 50 mol%) (Figure 3). The absolute concentrations (mmol/L) for total lipids and the main lipids in all the 14 lipoprotein subclasses are shown in Figure 5 (and in Supplementary Figure S5, available as Supplementary data at *IJE* online, for the NFBC86) together with a summary comparison between apolipoprotein B-containing particles and HDL particles. In absolute concentration terms, apolipoprotein B-containing particles transport roughly double the amount of cholesterol than HDL particles. The circulating pool of phospholipids is very similar in HDL and apolipoprotein B-containing particles, and very few triglycerides are transported in HDL.

### Associations between standard lipid and apolipoprotein measures

**Figure 6:**
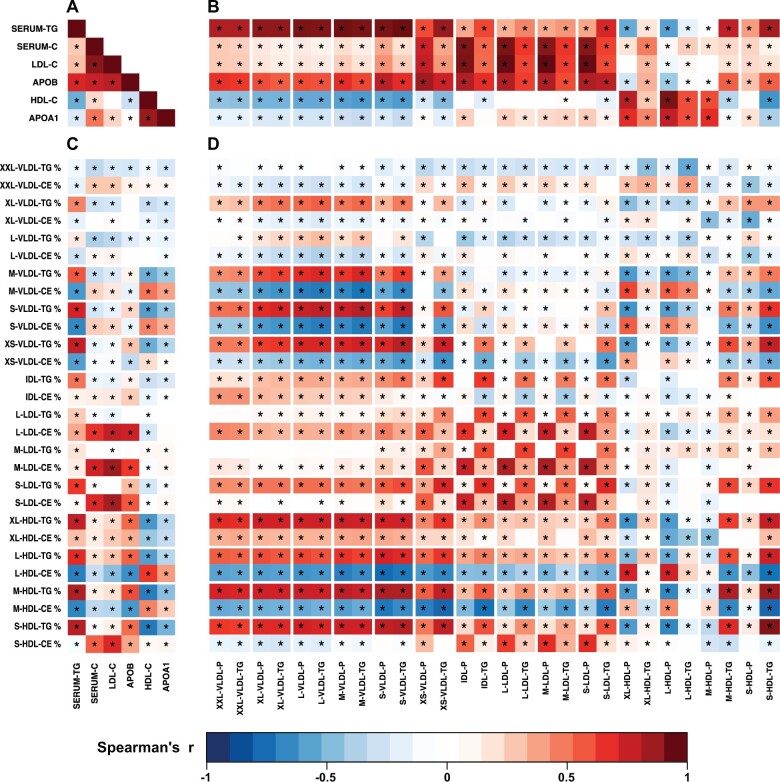
The associations between traditional lipoprotein lipid measures (A), between traditional lipoprotein lipid measures and lipoprotein subclass particle and triglyceride concentrations (B), between traditional lipoprotein lipid measures and lipoprotein subclass lipid composition for triglycerides and cholesteryl esters (C) and between lipoprotein subclass particle and triglyceride concentrations and lipoprotein subclass lipid composition for triglycerides and cholesteryl esters (D). The colour key refers to Spearman's rank correlation coefficients for the NFBC66 including 5651 participants (adjusted for sex). Twenty-three principal components explained >99% of the variation in the 174 lipoprotein measures (98 concentrations and 70 compositions for the lipoprotein subclasses, 4 traditional lipid measures and apolipoprotein A-I and B). Thus, a *P*-value threshold of 0.002 (i.e. 0.05/24) was used to denote evidence in favour of an association (marked * in the maps). The % sign refers to the compositional measures (i.e. the percentage of a lipid class concentration of the total lipid concentration in a particular lipoprotein subclass). Serum-TG, total circulating triglyceride concentration; Serum-C, total circulating cholesterol concentration; LDL-C, LDL cholesterol; APOB, apolipoprotein B; HDL-C, HDL cholesterol; APOA1, apolipoprotein A-I. Other abbreviations are as explained in the caption for Figure 1.

The standard lipid measures (also based on the NMR platform) behave as expected as illustrated in Figure 6A (and in Supplementary Figure S6A, available as Supplementary data at *IJE* online, for the NFBC86 data).^10^ The associations between apolipoprotein B and LDL cholesterol, total cholesterol and triglycerides are positive and strong. The negative association between HDL cholesterol and triglycerides is compelling. Apolipoprotein A-I is strongly positively associated with HDL cholesterol. Also, as expected, associations between apolipoprotein A-I and HDL particle concentrations and apolipoprotein B and the corresponding particle concentrations are strong (Supplementary Figure S7, available as Supplementary data at *IJE* online). On the other hand, associations between apolipoprotein A-I and B concentrations (g/L) as well as between HDL particle and apolipoprotein B-containing lipoprotein particle concentrations (mol/L) are very weak. This is explained by negative associations between apolipoprotein B and large and medium HDL particles and a counterbalancing effect due to a positive association between apolipoprotein B and the smallest HDL particles (Supplementary Figure S8, available as Supplementary data at *IJE* online).

### Associations between standard lipid measures and lipoprotein subclass concentrations

The associations between the 14 lipoprotein subclass measures and the standard lipid measures are all logical (Figure 6B and Supplementary Figure S6B, available as Supplementary data at *IJE* online). The associations of apolipoprotein B with all VLDL, IDL and LDL subclass concentration measures are positive and strong. VLDL subclass concentration measures associate positively with serum triglycerides and negatively with HDL cholesterol. Associations between LDL cholesterol and IDL and LDL subclass concentration measures are positive and strong. The associations with HDL subclass measures are heterogeneous, although HDL cholesterol and apolipoprotein A-I correlate positively with all HDL subclass particle concentrations.

### Associations between standard lipid measures and lipoprotein subclass compositions

The triglyceride composition of all lipoprotein subclasses is positively associated with the circulating triglyceride concentration, except in the case of XXL-VLDL (Figure 6C and Supplementary Figure S6C, available as Supplementary data at *IJE* online). The cholesteryl ester composition of all LDL subclass particles is strongly positively associated with circulating total and LDL cholesterol as well as apolipoprotein B concentrations. Circulating HDL cholesterol and apolipoprotein A-I concentrations are negatively associated with the triglyceride composition of all VLDL and IDL particles, positively associated with the cholesteryl ester composition of S-, M- and L-HDL particles, but the association is negative for XL-HDL particles.

### Associations between lipoprotein subclass concentrations and compositions

An opposite compositional behaviour is typical between triglycerides and cholesteryl esters **(**Figure 6D and Supplementary Figure S6D, available as Supplementary data at *IJE* online). For example, high circulating particle and triglyceride concentrations for apolipoprotein B-containing lipoprotein particles associate with triglyceride enrichment and diminished composition of cholesteryl esters in M- and L-HDL particles. Similar strong relationships exist between the concentrations as well as triglyceride and cholesteryl ester compositions of XS-, S-, M-, L- and XL-VLDL particles. The triglyceride composition of all HDL subclass particles is consistently positively associated with all apolipoprotein B-containing lipoprotein particle and triglyceride concentrations. The circulating concentrations of M-, L- and XL-HDL particles are negatively associated with their triglyceride compositions.

### Associations between lipoprotein subclass concentrations

**Figure 7:**
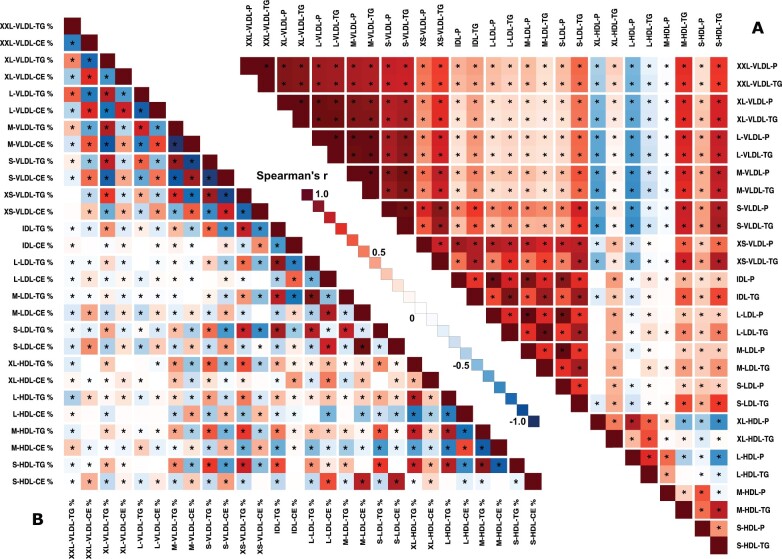
The associations between lipoprotein subclass particle and triglyceride concentrations (A) and between lipoprotein subclass lipid composition for triglycerides and cholesteryl esters (B). The data and abbreviations are as explained in the caption for Figure 6.

The associations between all the apolipoprotein B-containing lipoprotein subclass concentration measures form a coherent pattern and reflect the continuous metabolism from VLDL to LDL particles in the bloodstream (Figure 7A and Supplementary Figure S9A, available as Supplementary data at *IJE* online). Positive associations between adjacent subclasses are high and there is a general trend of weakening positive associations along the line of the metabolic direction from large VLDL to LDL particles. The association pattern appears different for XL- and L-HDL in comparison to M- and S-HDL subclasses. The subclass particle concentrations of large and medium HDL subclasses associate negatively with all VLDL subclasses but the associations for the smallest HDL subclass are in the opposite direction. Similarly, S-HDL particle concentrations are negatively associated with those of XL- and L-HDL. The circulating triglyceride concentration in every apolipoprotein B-containing subclass associates consistently positively with the corresponding measure in both M- and S-HDL.

### Associations between lipoprotein subclass compositions

The associations between lipoprotein subclass lipid compositions **(**Figure 7B and Supplementary Figure S9B, available as Supplementary data at *IJE* online) are generally a lot weaker and more heterogeneous than the corresponding associations between the concentration measures (Figure 7A and Supplementary Figure S9A, available as Supplementary data at *IJE* online). A global feature is that the triglyceride compositions of almost all lipoprotein subclass particles are strongly positively associated. These compositional triglyceride associations are strong in each major fraction of particles, i.e. within VLDL, IDL and LDL as well as HDL, but also mostly between these fractions with only a few triglyceride compositions in the largest VLDL subclasses showing either very weak or negative associations. Overall, triglyceride and cholesteryl ester compositions tend to associate negatively, particularly within the major fractions of particles.

A correlation heat map between all the lipoprotein subclass concentration, composition and standard lipid measures as well as apolipoprotein B and A-I are shown in Supplementary Figure S10 (available as Supplementary data at *IJE* online) for the NFBC66 and in Supplementary Figure S11 (available as Supplementary data at *IJE* online) for the NFBC86. The numerical data for these correlations are available in Supplementary Tables S2 and S3 (available as Supplementary data at *IJE* online), respectively. Supplementary Figures S12 and S13 (available as Supplementary data at *IJE* online) illustrate the comprehensive lipoprotein subclass data and the intertwined correlation structure in a metabolic network model.^27^ Another view on the correlation structure is given by the circular plots in Supplementary Figure S14 (available as Supplementary data at *IJE* online).

**Figure 8:**
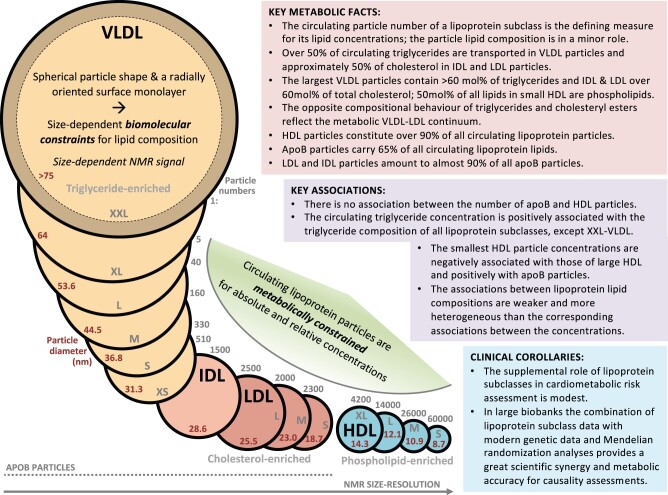
A summary figure encapsulating the key characteristics, associations and metabolic features of the 14 lipoprotein subclasses quantified with the NMR spectroscopy platform.^9^^,^^17^^,^^19^ The position of the individual lipoprotein subclass particles on the NMR size-resolution axis aims to picture the methodological resolution difference for large and small lipoprotein particles.^6^^,^^16^

Key results and the fundamental characteristics of lipoprotein subclass particles and metabolism are highlighted in a summary in Figure 8.

### Sex differences in lipoprotein subclass measures

Sex-specific data [mean (95% confidence interval range), *p*-value] in the NFBC66 for all the 168 lipoprotein subclass measures are given in Supplementary Table S4 (available as Supplementary data at *IJE* online) (composition measures) and Supplementary Table S5 (available as Supplementary data at *IJE* online) (concentration measures). Corresponding sex-specific data for a few clinically relevant lipoprotein measures are shown in Supplementary Table S6 (available as Supplementary data at *IJE* online). The current data replicate the well-known sex differences for clinical lipoprotein measures; females tend to have higher circulating HDL cholesterol and lower concentrations of apolipoprotein B-related measures.^28–31^ These overall differences are logically reflected at the lipoprotein subclass level. However, regarding HDL subclass particle concentrations, the main differences appear to be more pronounced for XL-HDL and L-HDL than for M-HDL. And, on the contrary, for S-HDL, the particle concentrations tend to be higher for males. Though females tend to have systematically less apolipoprotein B-containing particles in all the subclass categories, their relative lipid compositions are similar for males and females. However, males have consistently higher triglyceride compositions than females in all HDL subclass particles. The associations between the lipoprotein subclass measures are very similar for both sexes.

## Discussion

### Biomolecular and metabolic constraints regarding lipoprotein subclasses

It has been shown earlier that the circulating lipid concentrations as well as the relative lipid compositions for the lipoprotein subclasses with this methodology are very well concordant with those from density-based ultracentrifugation analyses,^4^^,^^32^ clinical chemistry^10^ and high-performance liquid chromatography.^17^ The compositional changes relative to triglycerides and cholesteryl esters are an intrinsic indication of the metabolic continuum in the VLDL–LDL metabolic cascade in the circulation.^13^^,^^33^^,^^34^ These reflect normal energy metabolism and the concerted actions of e.g. lipoprotein lipase (LPL), lecithin–cholesterol acyltransferase (LCAT) and cholesteryl ester transfer protein (CETP) in processing VLDL triglycerides and delivering free fatty acids to peripheral cells. HDL particles are likely to play a key role in these processes by reducing cholesterol influx into the arterial wall by accelerating the removal of atherogenic VLDL-related remnants from the circulation.^35^

As far as we are aware, the log-linear relationship between circulating lipoprotein subclass particle concentrations and the particle size has not been indicated before. The scale of difference in the subclass particle numbers is huge, e.g. 60 000 S-HDL particles per one circulating XXL-VLDL particle (Figure 8). On average, >90% of all circulating lipoprotein particles are HDL particles. Even though the particle volumes also differ considerably for different lipoprotein subclasses, the scale is far from the scale in the particle concentrations: the volume of an XXL-VLDL particle is ∼640 times that of an S-HDL particle. Therefore, the subclass particle concentrations are the main determinants of circulating lipoprotein subclass lipid concentrations and the variation in their lipid compositions is only a minor contributor. However, the view for circulating lipids is quite different from that of the individual particles: only 35% of all lipoprotein lipids are transported in HDL particles.

The key metabolic role of lipoprotein subclass particle concentrations is not unexpected. Within the particles, the size and structural molecular constraints fundamentally define how many non-polar and polar lipid molecules can be accommodated in both the core and the surface, respectively. Although the core and surface regions are not rigid, the spherical shape with an oriented monolayer poses strong biophysical constraints within the small number of key lipid classes—triglycerides, phospholipids, free and esterified cholesterol—to be integrated into the particles.^4^^,^^5^^,^^16^ Thus, functional metabolic issues, e.g. an increasing dietary fat load, must be solved by increasing the number of chylomicron (exogenously) and VLDL (endogenously) particles to transport the triglycerides; the structural modifications at the particle level would not be able to accommodate changes at that scale due to the biomolecular constraints. This does not mean, however, that the concentration issues would not affect the compositional characteristics (please see below in relation to triglycerides), but it is crucial to appreciate the largely different scale of achievable concentration changes and compositional modifications.

The log-linear relationship between lipoprotein subclass particle concentrations and the particle size also suggests the existence of fundamental metabolic constraints. This means not only that typical particle and lipid concentration ranges are elemental for humans, but also that only certain relative lipoprotein subclass particle concentrations are possible in human lipoprotein metabolism. We are not aware of previous discussion directly in relation to this, but we find it implicit in the lipoprotein literature and e.g. via the traditional clinical definitions of abnormalities in lipoprotein metabolism and their drug treatment.^36^ The large-scale data and the high resolution with respect to lipoprotein subclasses only amplify and clarify this issue. It is rather logical that a certain (even very high) load of nutritional fats (i.e. triglycerides) would lead to a corresponding number of transport particles, exogenously in the form of gut-derived chylomicron/apoB48-containing particles and endogenously via apoB100-containing VLDL particles.^33^ What can metabolically come out of the apolipoprotein B48/B100-containing lipoprotein cascades in the form of remnant, IDL and LDL subclass particles in the circulation must be in a direct relationship with the secreted number of chylomicron and/or VLDL particles. The number of HDL particles partly couples with this cascade, although also other sources and metabolic processes for HDL particles and their variation exist.^35^ Notably, apolipoprotein B and A-I concentrations (or apolipoprotein B-containing lipoprotein and HDL particle concentrations) only associate very weakly per se. This is explained by the opposite associations between apolipoprotein B and the larger/medium vs the smallest HDL particles.

Altogether, it is likely that these metabolic constraints are just simple reflections of normal metabolic pathways on how the human body handles triglycerides, cholesteryl esters and other dietary lipids. A key corollary, however, is that when evaluating a risk related to a lipoprotein subclass, it would only be relevant to estimate it within the physiologically realistic concentration area.^21–24^ For example, if risk calculations are done deviating from the physiological relationships, e.g. by calculating hazard ratios per an absolute concentration of circulating cholesterol (in mmol/l) in different lipoprotein fractions,^37^ misleading results are evident, as pointed out by Sniderman.^38^ Differences in hazard ratios are marked between 1 mmol/L and 1 SD cholesterol for IDL (5.48 vs 1.41 for myocardial infarction, respectively), since 1 SD for IDL cholesterol is only 0.20 mmol/L (i.e. 1 mmol/L changes for IDL cholesterol in a real population would be extremely rare and thus the risk calculated not representative). If 1 SD is close to the used absolute concentration difference, as in the case of non-HDL cholesterol (1 SD equates to 0.99 mmol/L), the calculated hazard ratios are similar too (1.48 vs 1.49, respectively).^39^ The appropriate approach would be to standardize the hazard ratios per standard deviation when aiming to compare the calculated risk for different lipoprotein measures—otherwise deceptive interpretations may result.^37–39^

### Standard lipids, lipoprotein subclasses and their lipid composition

The associations between the standard lipid measures and the subclass concentration measures are intuitive based on how the subclass particle categories are included in the standard lipid measures. For example, HDL cholesterol is the cholesterol carried in all the HDL subclass particles in the circulation and the triglyceride concentration refers to the sum of triglycerides in all the lipoprotein subclass particles. In the case of LDL cholesterol, the situation is clear within the NMR analysis, i.e. it represents the sum of the cholesterol in all LDL subclass particles. Thus, the LDL cholesterol based on the NMR platform is size-specific and does not include cholesterol in any other particles. Generally, however, ‘LDL’ as a concept is far from clear since multiple ‘LDL’ cholesterol assays exist based on remarkably varying methodologies, leading to between-assay heterogeneity in the values of ‘LDL’ cholesterol. The assay-dependent ambiguity can lead to either under- or overestimation of the true (size-specific) LDL cholesterol. We have recently discussed these issues and their potential implications in detail.^14^ The size-specific accurate definition of LDL and other lipoprotein subclasses is a definite virtue of the NMR-based lipoprotein analytics.

The overall association pattern for the lipoprotein subclass concentration measures is structured and characterized by strong correlations between adjacent apolipoprotein B-containing lipoprotein subclasses with clearly diminishing associations along the metabolic continuum from VLDL to LDL particles. On the other hand, the association pattern for lipoprotein subclass composition measures is varied and lacks pronounced patterns. Overall, triglyceride and cholesteryl ester compositions tend to associate negatively, particularly within the major lipoprotein fractions VLDL, IDL and LDL, and HDL. A consolidated interpretation of all the associations between the concentration and composition measures is the consistent behaviour of triglycerides. High circulating triglyceride concentrations are reflected by high concentrations of triglyceride-enriched VLDL particles that appear to cause a metabolic spillover effect and result consistently in triglyceride-enriched particles in all lipoprotein subclasses. The smallest HDL subclass behaves similarly to apolipoprotein B-containing subclasses regarding various associations, in contradiction to the other HDL subclasses.

### Large-scale epidemiology in relation to lipoprotein subclass data

This particular NMR platform has been applied in a few large-scale prospective cardiovascular outcome studies with the focus on lipoprotein subclass concentrations.^21–24^^,^^40^ The lipoprotein subclass risk profile for cardiovascular events in a European population^21^ and for incident myocardial infarction in a Chinese population^22^ appears highly concordant. The subclass risk profile is similar between incident myocardial infarction and ischaemic stroke, but differs considerably for intracerebral haemorrhage for which none of the lipoprotein subclasses appears as a risk factor.^22^ There are also apparent differences in how the lipoprotein subclasses associate with peripheral and coronary artery disease, the associations being weaker for the former.^24^ Applications in other areas, as reviewed by Würtz *et al*. in 2017,^10^ have also almost entirely utilized only lipoprotein subclass concentration data.^10^ Most recent large-scale applications involve studies on e.g. oral glucose tolerance test,^41^ healthy diets and risk of cardiovascular disease,^42^ and exome sequencing for rare variants in cardiometabolic traits.^43^

### Large-scale epidemiology in relation to compositional data on lipoprotein subclasses

Only a few large-scale epidemiological studies have exploited the compositional lipoprotein subclass data from this NMR platform or in general. Laakso and co-workers studied lipoprotein subclass profiles in individuals with varying degrees of glucose tolerance in a population-based study of >9000 Finnish men.^20^ Concomitantly to increased serum concentrations of essentially all apolipoprotein B-containing lipoprotein particles as well as the smallest HDL particles, hyperglycaemia and insulin resistance were associated with triglyceride enrichment in various subclass particles, although these differences were related more to hypertriglyceridaemia than to hyperglycaemia.^20^ Barrios *et al.*^44^ noted that in both diabetic and non-diabetic individuals, the triglyceride composition of various apolipoprotein B-containing and some HDL particles were rather consistently inversely associated with kidney function and microvascular complications of diabetes. A recent study examined observational associations of all lipoprotein subclass measures with risk of incident CHD in three population-based cohorts totalling 616 incident cases and 13 564 controls during 8-year follow-up.^13^ This study was the first large-scale study to assess the independent role of lipoprotein subclass composition in CHD risk assessment with the finding that a higher triglyceride composition within HDL subclasses was associated with higher risk of CHD, independently of total cholesterol and triglycerides. The largest hazard ratio for the triglyceride enrichment in medium HDL subclass particles was of a similar magnitude as that for LDL cholesterol and apolipoprotein B, i.e. ∼1.3. However, the findings were interpreted to reflect the combined effects of circulating HDL and apolipoprotein B-containing particles, possibly in connection with CETP function and the circulating number of total triglycerides, not an intrinsic indication of an independent role of HDL particle lipid composition in CHD.^13^

### Drug-target Mendelian randomization

The lipoprotein subclass data have recently been actively utilized in drug-target Mendelian randomization analyses.^12^^,^^13^^,^^15^^,^^45^^,^^46^ In these studies, specific genetic instruments are used as a proxy for the pharmacological on-target effects of proteins to understand and quantify the involvement of a protein in disease.^45–47^ The detailed size resolution provided by the NMR-based lipoprotein data has been advantageous in the detailed appreciation of the intricate details and differences in various cholesterol-lowering drugs.^12^^,^^15^^,^^45^ The comparison between the results from actual randomized–controlled drug trials and drug-target Mendelian randomization is compelling, as recently demonstrated in a prospective pravastatin trial together with comparisons to the metabolic effects of genetic inhibition of HMG-CoA reductase (statins) and proprotein convertase subtilisin/kexin type 9 (PCSK9).^12^ A drug-target Mendelian randomization study on CETP inhibition revealed that conventional composite lipid assays (e.g. ‘LDL cholesterol’)^14^ may mask important heterogeneous effects on lipoprotein profiles.^13^^,^^48^ The differences between the observed drug effects on traditional ‘LDL cholesterol’ and, both analytically and metabolically, more specific apolipoprotein B-containing lipoprotein subclasses are calling attention to the need for metabolic precision in the measurements of lipoprotein data and in assessing the role of lipoprotein metabolism in cardiovascular disease in relation to existing and novel lipid-altering therapies. A particularly problematic issue would be to use a non-specific marker of lipids to derive an expected effect of a drug with risk of disease.^13^^,^^14^ The combination of NMR-based lipoprotein subclass data with drug-target Mendelian randomization analyses has shown a great synergy for drug development in the current era of biobanks and big data. However, it should be kept in mind that the use of drug-target Mendelian randomization analysis can principally provide information only on the pharmacological on-target effects (of a class of drugs) and does not allow the assessment of potential off-target effects (of individual drugs).^49^

### Lipoprotein subclass measures as risk factors

The high size resolution offered by NMR-based lipoprotein profiling has resulted in a detailed understanding of how the various stages and remnant particles in the apolipoprotein B-containing lipoprotein cascade relate to cardiometabolic issues and outcomes.^10^^,^^21^^,^^22^^,^^34^^,^^50^ However, from the predictive-risk-factor perspective, the additional information provided by the lipoprotein subclass particle concentrations in common cardiovascular disease outcomes appears inconsequential, particularly keeping in mind the current recognition of the key role of apolipoprotein B in the development of atherosclerosis and CHD.^13^^,^^21^^,^^51–53^ The potential role of lipoprotein particle compositions (independent of concentrations) has occasionally been discussed in the literature since the advent of sequential preparative ultracentrifugation for lipoprotein analytics, but the tediousness and cost of the process have precluded large-scale studies.^2^^,^^13^ The clinically most utilized NMR-based lipoprotein analytics unfortunately does not provide compositional lipoprotein subclass data.^7^^,^^54^ Thus, the first large-scale study on the independent role of lipoprotein particle compositions on CHD was published only very recently and based on the NMR methodology under scrutiny here.^13^ However, as discussed above, the most pronounced findings regarding the increased risk in relation to triglyceride enrichment of HDL particles might just be a reflection of high circulating triglycerides and not an intrinsic indication of an independent role of HDL particle lipid composition in CHD.^13^ Nonetheless, in type 1 diabetes, triglyceride–cholesterol imbalance has been found as a general lipoprotein characteristic indicating high vascular disease risk.^55^

## Limitations

The separation of lipoprotein subclasses in NMR is solely based on particle size. Fundamentally, the resolution via NMR chemical shifts is best for the smallest particles and weakest for the largest particles (Figure 8).^16^ In postprandial conditions, large chylomicron particles will be detected in the largest-particle category XXL-VLDL without any possibility of directly resolving VLDL and chylomicron particles. In addition, if smaller apoB48-containing particles are secreted by the gut into the bloodstream,^33^ they are detected purely based on their particle size without any possibility of further identification. This limitation applies also to all potential remnant particles; the detection is based only on the particle size and further metabolic identification, e.g. between a VLDL or a chylomicron remnant, is not possible, i.e. all will be summed up together according to their particle size.

The highly correlated nature of lipoprotein subclass concentration measures, particularly for adjacent subclasses, is sometimes identified as a limitation or a problem. However, this is a direct representation of human lipoprotein metabolism; it is a continuum, particularly for the apolipoprotein B-containing lipoprotein subclasses. Thus, the adjacent subclass particles, in both concentrations and composition, are inevitably very similar with highly correlated attributes. Particularly at high size resolution of the particles, any other correlation behaviour between measures for adjacent or closely related lipoprotein subclasses would most likely be an indication of methodological hurdles. Nevertheless, the high correlations make part of the information redundant from a strictly statistical perspective but, on the other hand, in the case of continuous metabolic processes, high resolution allows intricate interpretations and findings as already demonstrated in various publications.^10^^,^^12^^,^^13^^,^^15^^,^^34^^,^^45^

## Conclusions

The various analyses presented here reveal fundamental characteristics and differences in lipoprotein subclass concentration and composition data. We would urge clear nomenclature in relation to these measures and noting consistently whether circulating particle (mol/L) or lipid concentrations (mmol/L) are meant or whether referring to a relative lipid composition in a particular particle subclass (mol% of total lipids). For example, a triglyceride measure could refer to a circulating concentration in the bloodstream of a particular subclass of particles (mmol/L) or to a relative composition of triglycerides in a particular particle subclass (mol% of total lipids). These measures have a very different biological meaning and interpretation. The overall spherical structure of these particles, with an oriented surface monolayer, results in strong size-dependent biomolecular constraints for their lipid compositions. In addition, the human lipoprotein metabolism appears to be fundamentally controlled in such a way that only certain lipoprotein subclass particle and lipid concentrations are possible in the circulation. Lipoprotein subclass-related risk analyses are thus relevant only within physiologically feasible conditions. Comprehensive data on lipoprotein subclass particles in large-scale studies provide opportunities for detailed understanding of lipoprotein metabolism in health and disease. Combining these data with modern genetics in exceedingly extensive studies as in the UK Biobank will facilitate grasping what features might represent causal, and thus potentially treatable and druggable, metabolic risk factors.

## Supplementary data

Supplementary data are available at *IJE* online.

## Ethics approval

The studies comply with the Declaration of Helsinki, were approved by the local ethics committee (Northern Ostrobothnia Hospital District, Finland) and written informed consents were obtained from each participant.

## Funding

M.A.K. is supported by a research grant from the Sigrid Juselius Foundation, Finland. P.O. is supported by the Emil Aaltonen Foundation. The Northern Finland Birth Cohorts have received funding from the Academy of Finland, Novo Nordisk Foundation and EU.

## Data availability

The NFBC data sets are available through an application process for researchers who meet the criteria for access to confidential data. Please contact the project centre (NFBCprojectcenter@oulu.fi) and visit the website (www.oulu.fi/nfbc) for more information.

## Conflict of interest

None declared.

## Supplementary Material

dyab156_Supplementary_Data
