# Supplementary material for: Apt interpretation of comprehensive lipoprotein data in large-scale epidemiology: disclosure of fundamental structural and metabolic relationships
Source: Int J Epidemiol. 2021 Aug 18;51(3):996–1011. doi: 10.1093/ije/dyab156 (PMC9189959; doi:10.1093/ije/dyab156)
Supplement: dyab156_Supplementary_Data [file dyab156_supplementary_data.zip › ije-2021-01-0073-File010.pdf]

Supplemental material for:

Apt interpretation of comprehensive lipoprotein data in large-scale epidemiology – disclosure of fundamental structural and metabolic relationships

Mika Ala-Korpela, Siyu Zhao, Marjo-Riitta Järvelin,
Ville-Petteri Mäkinen, and Pauli Ohukainen

Supplement content

Supplement Note

Study populations

Supplement Tables

Table S1. Clinical characteristics for the NFBC66 and NFBC86 cohorts (median [interquartile range]).

Table S2. (Excel) Spearman's rank correlations (adjusted for sex) for the lipoprotein subclass concentration (98), composition (70) and standard lipid (4) measures as well as apolipoprotein B and A-I for the 5,651 participants in NFBC66 (Figure S10).

Table S3. (Excel) Spearman's rank correlations (adjusted for sex) for the lipoprotein subclass concentration (98), composition (70) and standard lipid (4) measures as well as apolipoprotein B and A-I for the 5,605 participants in NFBC86 (Figure S11).

Table S4. (Excel) Sex specific data (mean [95% CI range], p-value) for lipoprotein subclass composition measures in NFBC66.

Table S5. (Excel) Sex specific data (mean [95% CI range], p-value)) for lipoprotein subclass concentration measures in NFBC66.

Table S6. Sex specific data for a few clinically relevant lipoprotein measures in NFBC66 (median [interquartile range], p-value).

Supplement Figures

Figure S1. The relative lipid compositions of the 14 lipoprotein subclasses for the 5,605 participants in NFBC86.

Figure S2. The circulatory and compositional characteristics of lipoprotein subclasses for the 5,605 participants in NFBC86.

Figure S3. A. The distributions of particle concentrations for each lipoprotein subclass for the 5,605 participants in NFBC86. **B.** Various proportions of lipoprotein particles and lipids for the 5,605 participants in NFBC86.

Figure S4. The log-linear relationship between the circulating lipoprotein subclass particle concentration and the particle diameter (5,605 participants in NFBC86).

Figure S5. The absolute circulating lipid concentrations for each lipoprotein subclass and the corresponding summary measures for the apolipoprotein B-containing lipoprotein particles and HDL particles for the 5,605 participants in NFBC86.

Figure S6. For the 5,605 participants in NFBC86: **A.** The associations between traditional lipoprotein lipid measures. **B.** The associations between traditional lipoprotein lipid measures and lipoprotein subclass particle and triglyceride concentrations. **C.** The associations between traditional lipoprotein lipid measures and lipoprotein subclass lipid composition for triglycerides and cholesteryl esters. **D.** The associations between lipoprotein subclass particle and triglyceride concentrations and lipoprotein subclass lipid composition for triglycerides and cholesteryl esters.

Figure S7. Scatter plots for the 5,651 participants in NFBC66: **A.** Apolipoprotein B concentration vs apolipoprotein B particle concentration. **B.** Apolipoprotein A-I concentration vs HDL particle concentration. **C.** Apolipoprotein B concentration vs apolipoprotein A-I concentration. **D.** Apolipoprotein B particle concentration vs HDL particle concentration.

Figure S8. Scatter plots for the 5,651 participants in NFBC66: **A.** Apolipoprotein B particle concentration vs XL-HDL particle concentration. **B.** Apolipoprotein B particle concentration vs L-HDL particle concentration. **C.** Apolipoprotein B particle concentration vs M-HDL particle concentration. **D.** Apolipoprotein B particle concentration vs S-HDL particle concentration.

Figure S9. For the 5,605 participants in NFBC86: **A.** The associations between lipoprotein subclass particle and triglyceride concentrations. **B.** The associations between lipoprotein subclass lipid composition for triglycerides and cholesteryl esters.

Figure S10. A heatmap of Spearman's rank correlations (adjusted for sex) for the lipoprotein subclass concentration (98), composition (70) and standard lipid (4) measures as well as apolipoprotein B and A-I for the 5,651 participants in NFBC66 (Table S1).

Figure S11. A heatmap of Spearman's rank correlations (adjusted for sex) for the lipoprotein subclass concentration (98), composition (70) and standard lipid (4) measures as well as apolipoprotein B and A-I for the 5,605 participants in NFBC86 (Table S2).

Figure S12. A Spearman's rank correlation network between lipoprotein subclasses for the 5,651 participants in NFBC66. All correlations with $R^2 > 10\%$ are shown in the plot.

Figure S13. A Spearman's rank correlation network between lipoprotein subclasses for the 5,651 participants in NFBC66. A pruned version of the network show in Figure S12.

Figure S14. Circular visualisations of the strongest Spearman's rank correlations **A.** between lipoprotein subclasses and apolipoprotein A-I and B and **B. – O.** between lipoprotein subclasses for the 5,651 participants in NFBC66.

Supplement Note

Study populations

The Northern Finland Birth Cohort (NFBC) studies are two longitudinal birth cohorts established to study factors affecting preterm birth and consequent morbidity in the two northernmost provinces of Finland, Oulu and Lapland. The NFBC66 includes 12,058 live births (12,231 children) covering 96% of all eligible births in this region during January – December 1966.[1] The participants were followed-up at the age of 1, 14, 31 and 46 years. Data collection conducted in 2012 at their age of 46 years, including clinical examination and fasting serum sampling and NMR spectroscopy, was available for 5,651 participants and these data were used for the main analyses in this work. Two decades later, a second cohort of 9,432 births was collected (NFBC86) which covered 99% of all the deliveries taking place in the target regions during July 1985 – June 1986.[2] Data collection in 2001–2002 including clinical examination, fasting serum samples and NMR data at the age of 15–16 was available for 5,605 adolescents and was used as a replication in this work. The studies comply with the Declaration of Helsinki, were approved by the local ethics committee (Northern Ostrobothnia Hospital District, Finland) and written informed consents were obtained from each participant.

References

1. Sabatti C, Service SK, Hartikainen A-L, Pouta A, Ripatti S, Brodsky J, Jones CG, Zaitlen NA, Varilo T, Kaakinen M, Sovio U, Ruokonen A, Laitinen J, Jakkula E, Coin L, Hoggart C, Collins A, Turunen H, Gabriel S, Elliot P, McCarthy MI, Daly MJ, Järvelin M-R, Freimer NB, Peltonen L. Genome-wide association analysis of metabolic traits in a birth cohort from a founder population. *Nat Genet* 2009;**41**:35–46.

2. Nedelec R, Miettunen J, Männikkö M, Järvelin M-R, Sebert S. Maternal and infant prediction of the child BMI trajectories; studies across two generations of Northern Finland birth cohorts. *Int J Obes (Lond)* 2020;

3. Mäkinen V-P, Tynkkynen T, Soininen P, Peltola T, Kangas AJ, Forsblom C, Thorn LM, Kaski K, Laatikainen R, Ala-Korpela M, Groop P-H. Metabolic Diversity of Progressive Kidney Disease in 325 Patients with Type 1 Diabetes (the FinnDiane Study). *J Proteome Res* 2012;**11**:1782–1790.

Supplement Tables

Table S1. Clinical characteristics for the NFBC66 and NFBC86 cohorts (median [interquartile range]).

	**NFBC66**	**NFBC86**
Participants (female %)	5651 (56.2%)	5604 (49.6%)
Age (years)	46.6 [46.2 - 47.1]	16.0 [15.8 - 16.3]
BMI (kg/m^2)	26.1 [23.5 - 29.3]	20.5 [19.0 - 22.6]
Waist-hip ratio	0.91 [0.85 - 0.98]	0.80 [0.76 - 0.83]
Systolic BP (mmHg)	124 [114 - 135]	115 [107 - 124]
Diastolic BP(mmHg)	84 [77 - 92]	68 [63 - 73]
Glucose (mmol/L)	4.8 [4.5 - 5.1]	4.5 [4.2 - 4.8]
Triglycerides (mmol/L)	1.1 [0.8 - 1.6]	0.9 [0.7 - 1.1]
LDL cholesterol (mmol/L)	2.2 [1.8 - 2.6]	1.5 [1.2 - 1.8]
HDL cholesterol (mmol/L)	1.6 [1.4 - 1.9]	1.4 [1.2 - 1.6]

For Tables S2 – S5 see an attached Excel file.

Table S6. Sex specific data for a few clinically relevant lipoprotein measures in NFBC66 (median [interquartile range], p-value).

	**Male**	**Female**	**p-value**
Triglycerides (mmol/L)	1.4 [1.0 - 1.9]	1.0 [0.7 - 1.3]	9.8E-108
Total cholesterol (mmol/L)	5.8 [5.1 - 6.5]	5.5 [4.9 - 6.2]	1.0E-19
LDL cholesterol (mmol/L)	2.4 [1.9 - 2.8]	2.1 [1.7 - 2.4]	2.0E-63
HDL cholesterol (mmol/L)	1.4 [1.2 - 1.7]	1.8 [1.5 - 2.1]	2.3E-218
Apolipoprotein B (g/L)	1.1 [1.0 - 1.3]	1.0 [0.8 - 1.1]	7.3E-143
Apolipoprotein A-I (g/L)	1.7 [1.5 - 1.8]	1.8 [1.6 - 1.9]	5.2E-108

Supplement Figures

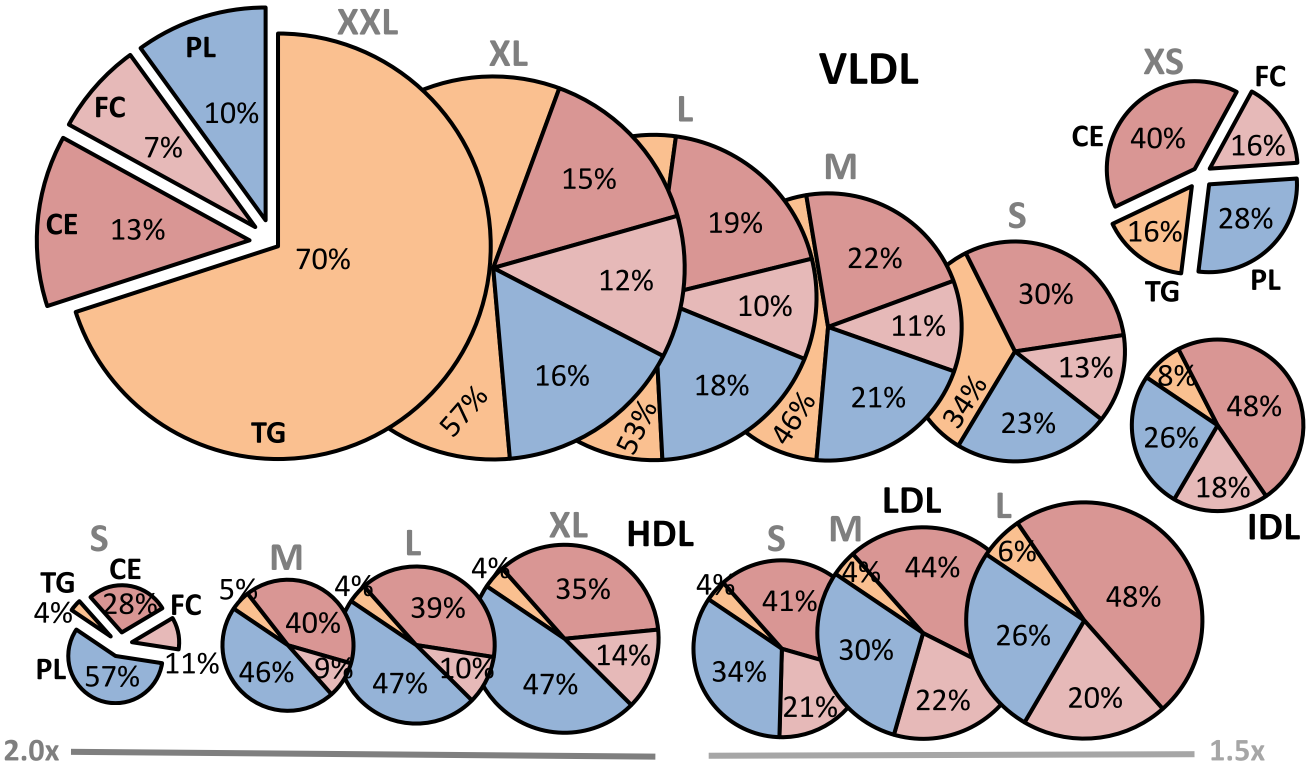

Figure S1

The relative lipid compositions of the 14 lipoprotein subclasses. The NMR platform resolution is 14 lipoprotein subclasses, defined by their particle size as follows: six VLDL particle categories; XXL-VLDL (with average particle diameter >75 nm), XL-VLDL (64 nm), L-VLDL (53.6 nm), M-VLDL (44.5 nm), S-VLDL (36.8 nm), and XS-VLDL (31.3 nm); IDL (28.6 nm), L-LDL (25.5 nm), M-LDL (23.0 nm), and S-LDL (18.7 nm); and XL-HDL (14.3 nm), L-HDL (12.1 nm), M-HDL (10.9 nm) and S-HDL (8.7 nm). Note that the size of LDL and HDL particles in the figure is multiplied by 1.5 and 2.0, respectively. The data are mean values for 5,605 participants in NFBC86. VLDL, very-low-density lipoprotein; IDL, intermediate-density lipoprotein; LDL, low-density lipoprotein; HDL, high-density lipoprotein; XXL, extremely large; XL, very large; L, large; M, medium; S, small; XS, very small; TG, triglycerides; PL, phospholipids; CE, cholesteryl esters; FC, free cholesterol.

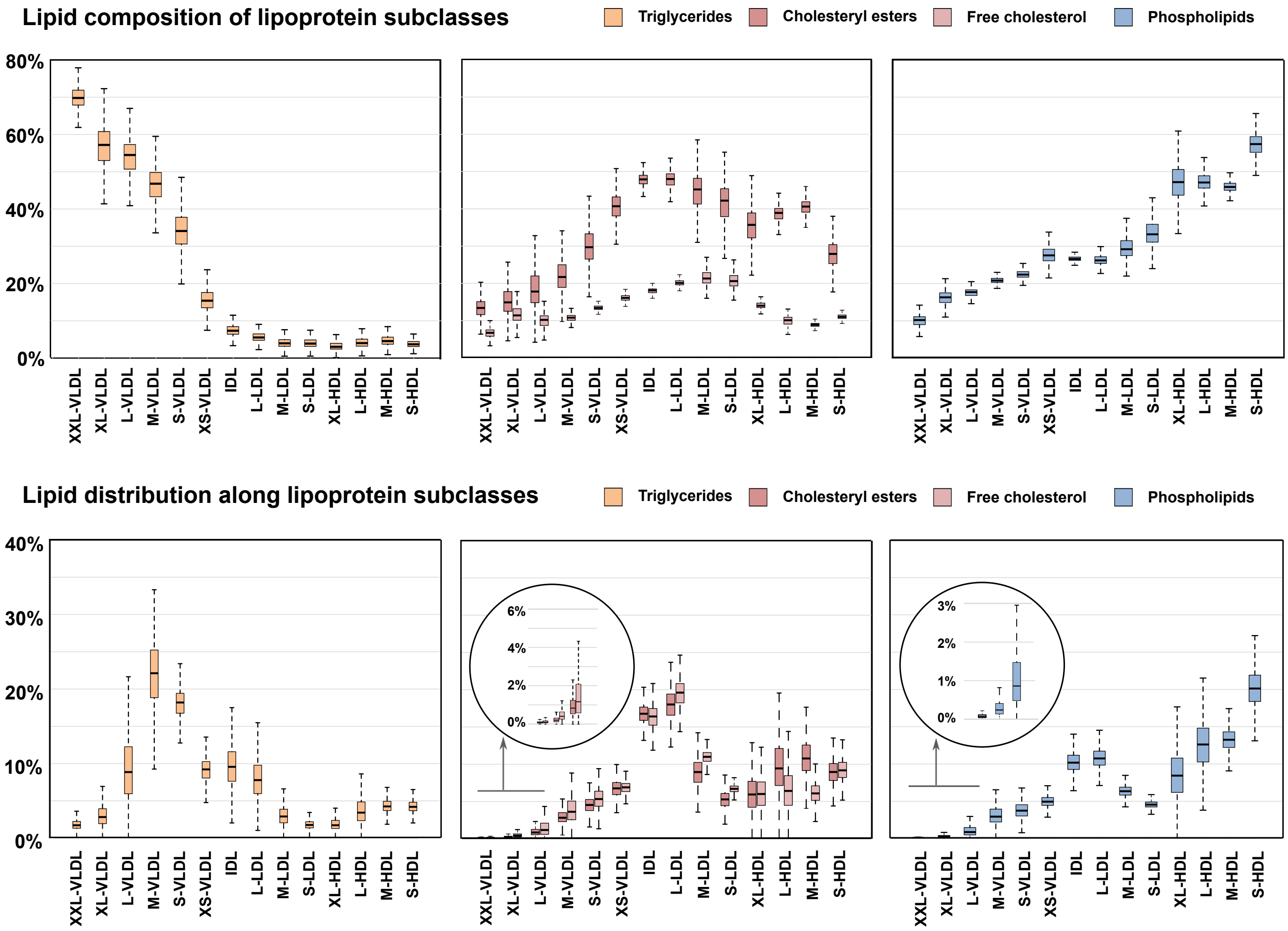

Figure S2

The circulatory and compositional characteristics of lipoprotein subclasses. The lipid distribution along the lipoprotein subclasses illustrates how a certain lipid class is distributed in the blood stream among all the lipoprotein subclasses (i.e., the sum of all percentages in each inset is 100%). The composition of lipoprotein subclasses depicts what are the relative lipid contents in each subclass particle category (i.e., the sum of the percentages of all the lipid classes for each subclass is 100%). The data are from the NFBC86 including 5,605 participants; each box plot shows the median within the interquartile range (IQR) and the minimum (Q1 - 1.5*IQR) and maximum (Q3 + 1.5*IQR) values with potential outliers. The abbreviations are as explained in the caption for Figure S1.

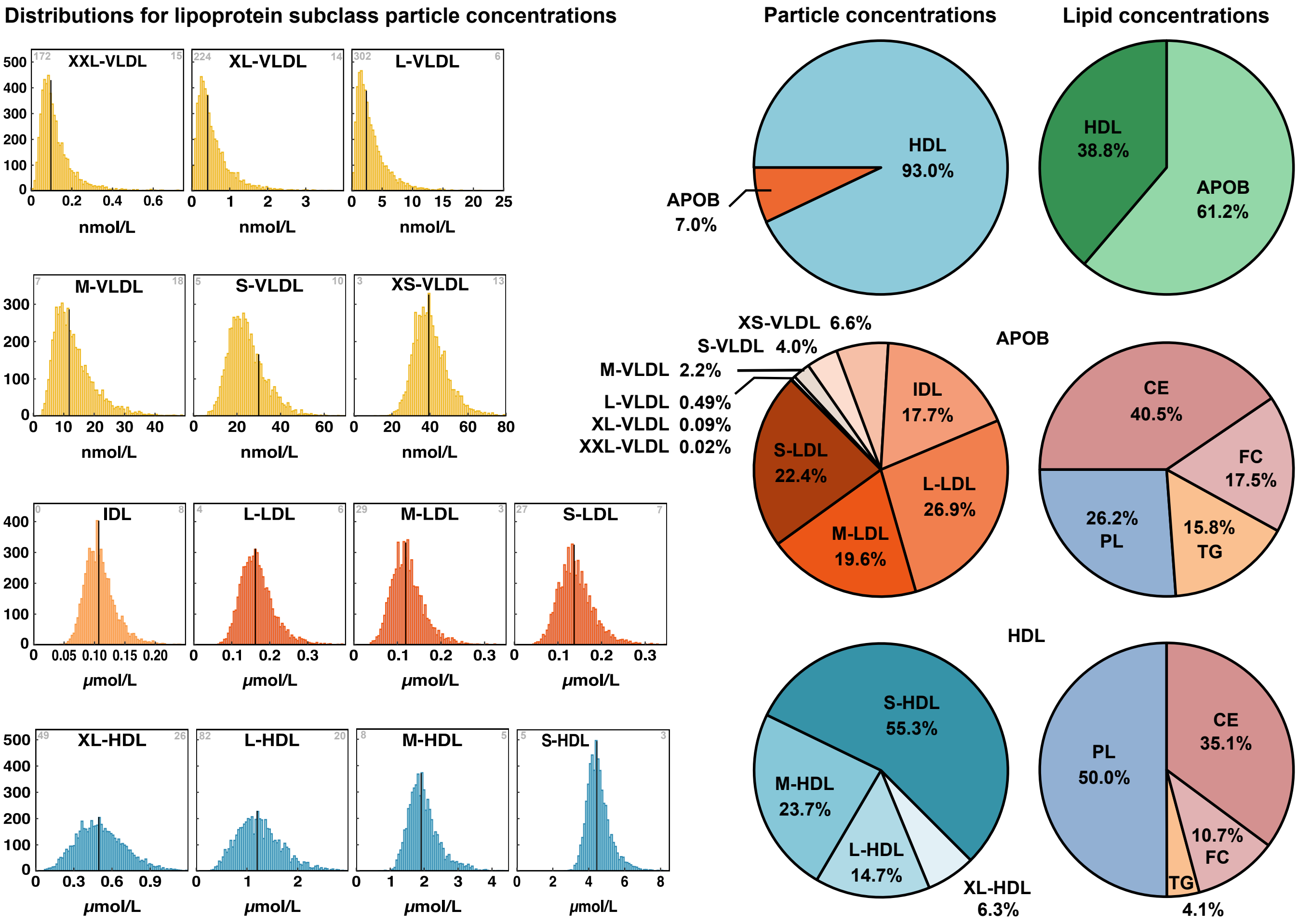

Figure S3

The distributions of particle concentrations for each lipoprotein subclass. The data are from the NFBC86 including 5,605 participants. The grey number in the upper-left corner identifies the number of samples for which the particle concentration is zero and in the upper-right corner for how many high concentration values were cut off from the drawn distribution. Note that the concentration scale is nmol for the VLDL particles and μmol for the IDL, LDL and HDL particles. The black vertical lines denote the median concentration values. Various proportions of lipoprotein particles and lipids are shown in the pie charts as mean values for the 5,605 participants in NFBC86. The abbreviations are as explained in the caption for Figure S1.

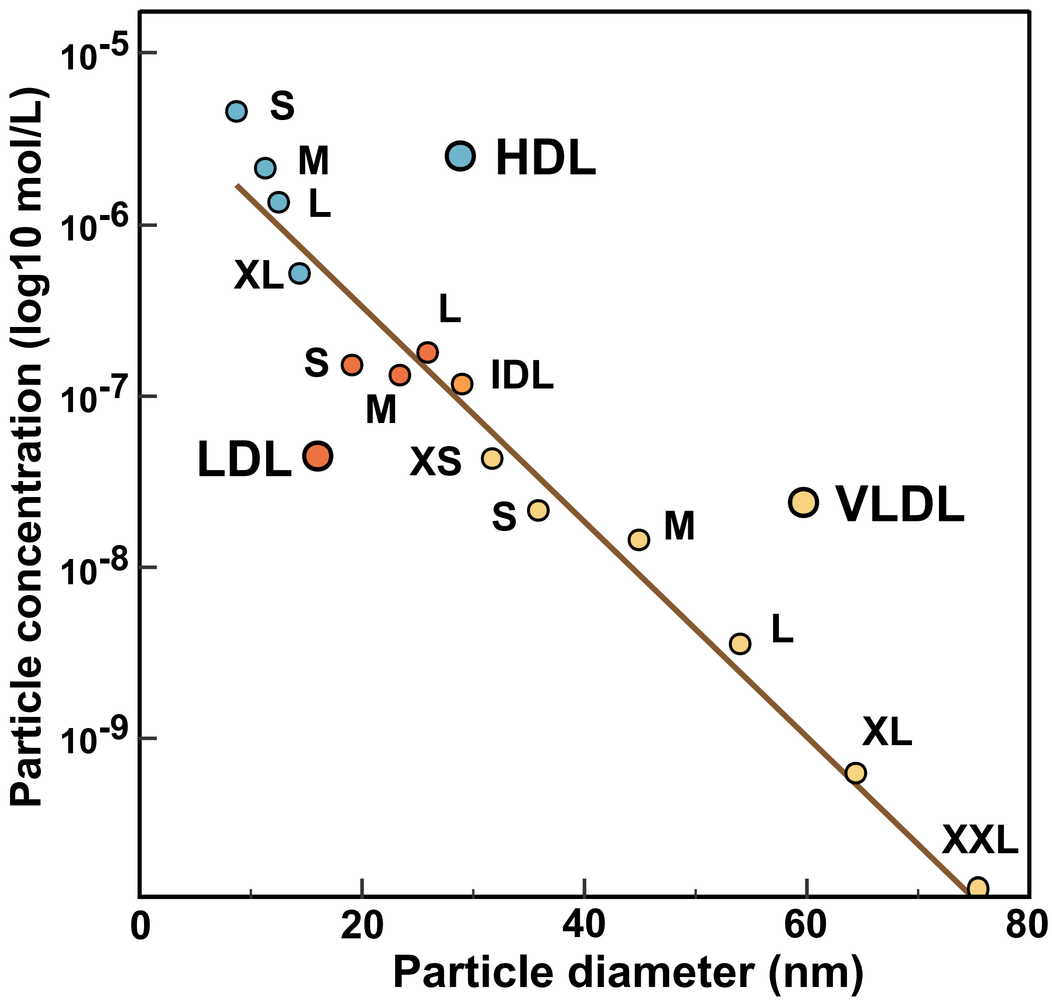

Figure S4

The log-linear relationship between the circulating lipoprotein subclass particle concentration and the particle diameter. The data and abbreviations are as explained in the caption for Figure S1.

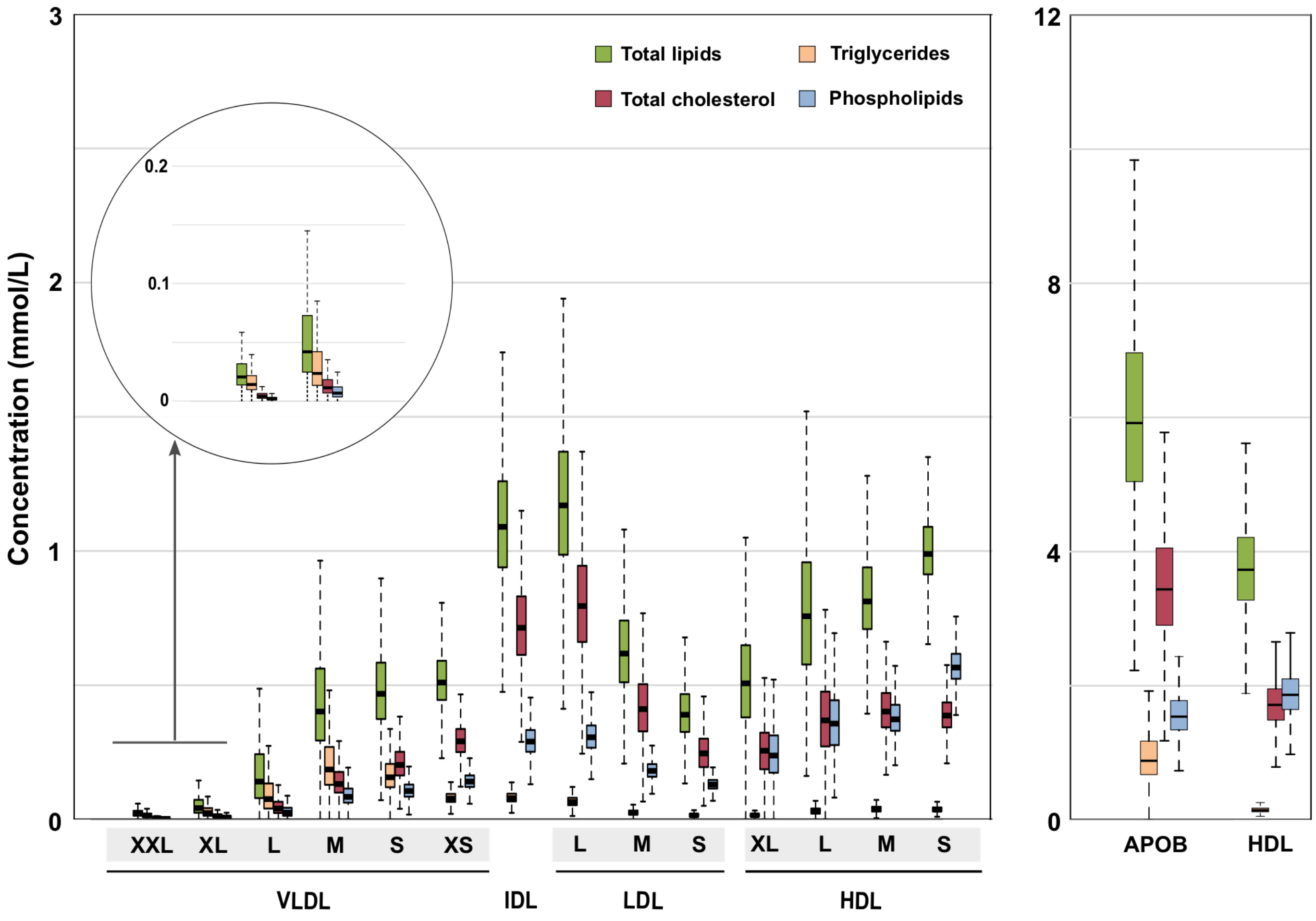

Figure S5

The absolute circulating lipid concentrations for each lipoprotein subclass and the corresponding summary measures for the apolipoprotein B-containing lipoprotein particles and HDL particles. The data and abbreviations are as explained in the captions for Figure S1 and S2.

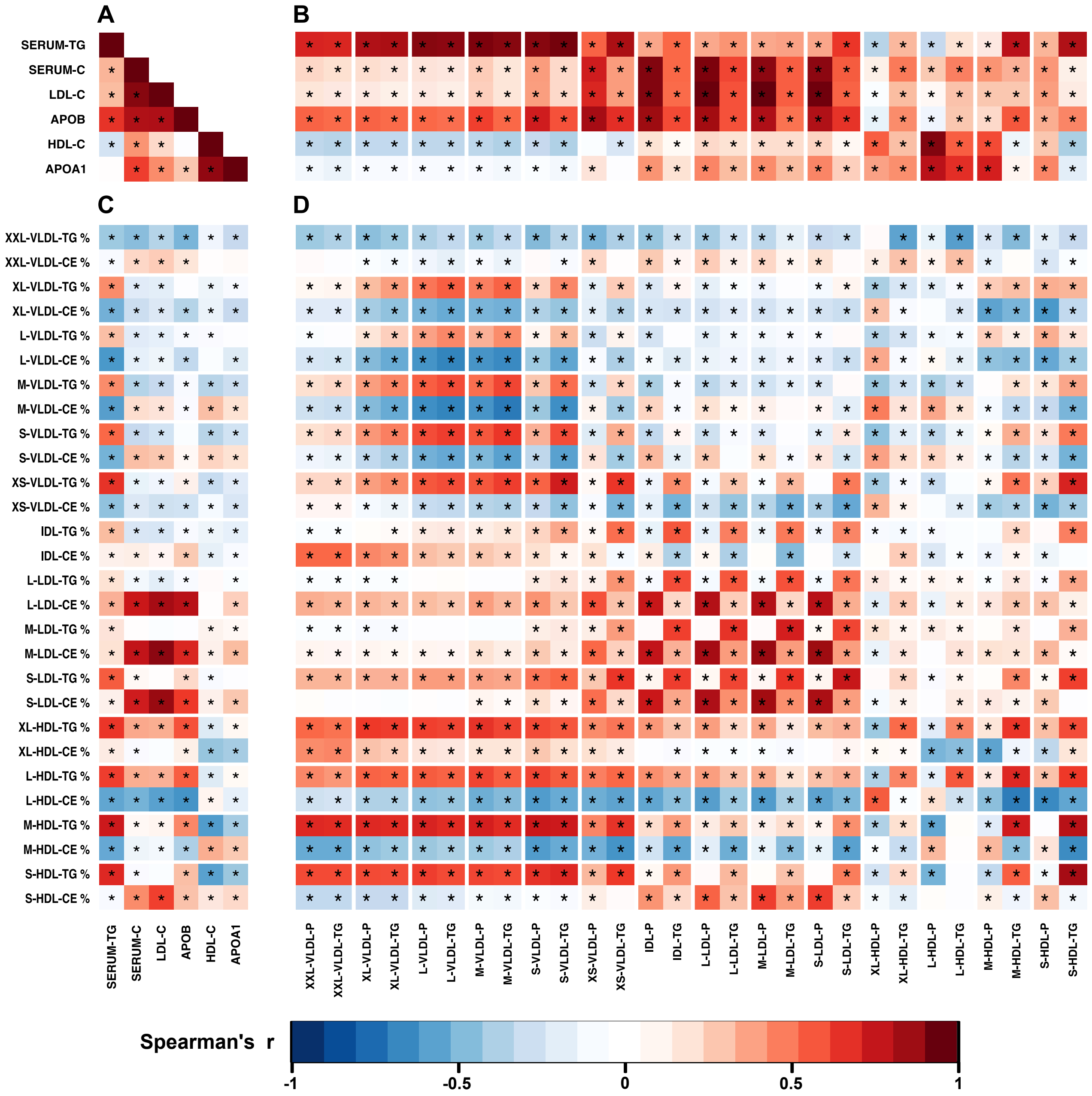

Figure S6

The associations between traditional lipoprotein lipid measures (A), between traditional lipoprotein lipid measures and lipoprotein subclass particle and triglyceride concentrations (B), between traditional lipoprotein lipid measures and lipoprotein subclass lipid composition for triglycerides and cholesteryl esters (C), and between lipoprotein subclass particle and triglyceride concentrations and lipoprotein subclass lipid composition for triglycerides and cholesteryl esters (D). The colour key refers to Spearman's rank correlation coefficients the NFBC86 including 5,605 participants (adjusted for sex). Twenty-three

principal components explained >99% of variation in the 174 lipoprotein measures (98 concentrations and 70 compositions for the lipoprotein subclasses, 4 traditional lipid measures and apolipoprotein A-I and B). Thus, a P-value threshold of 0.002 (*i.e.*, 0.05/25) was used to denote evidence in favour of an association (marked * in the maps). The %-sign refers to the compositional measures (i.e., the percentage of a lipid class concentration of the total lipid concentration in a particular lipoprotein subclass). Serum-TG, total circulating triglyceride concentration; Serum-C, total circulating cholesterol concentration; LDL-C, LDL cholesterol; APOB, apolipoprotein B; HDL-C, HDL cholesterol; APOA-I, apolipoprotein A-I. Other abbreviations are as explained in the caption for Figure S1.

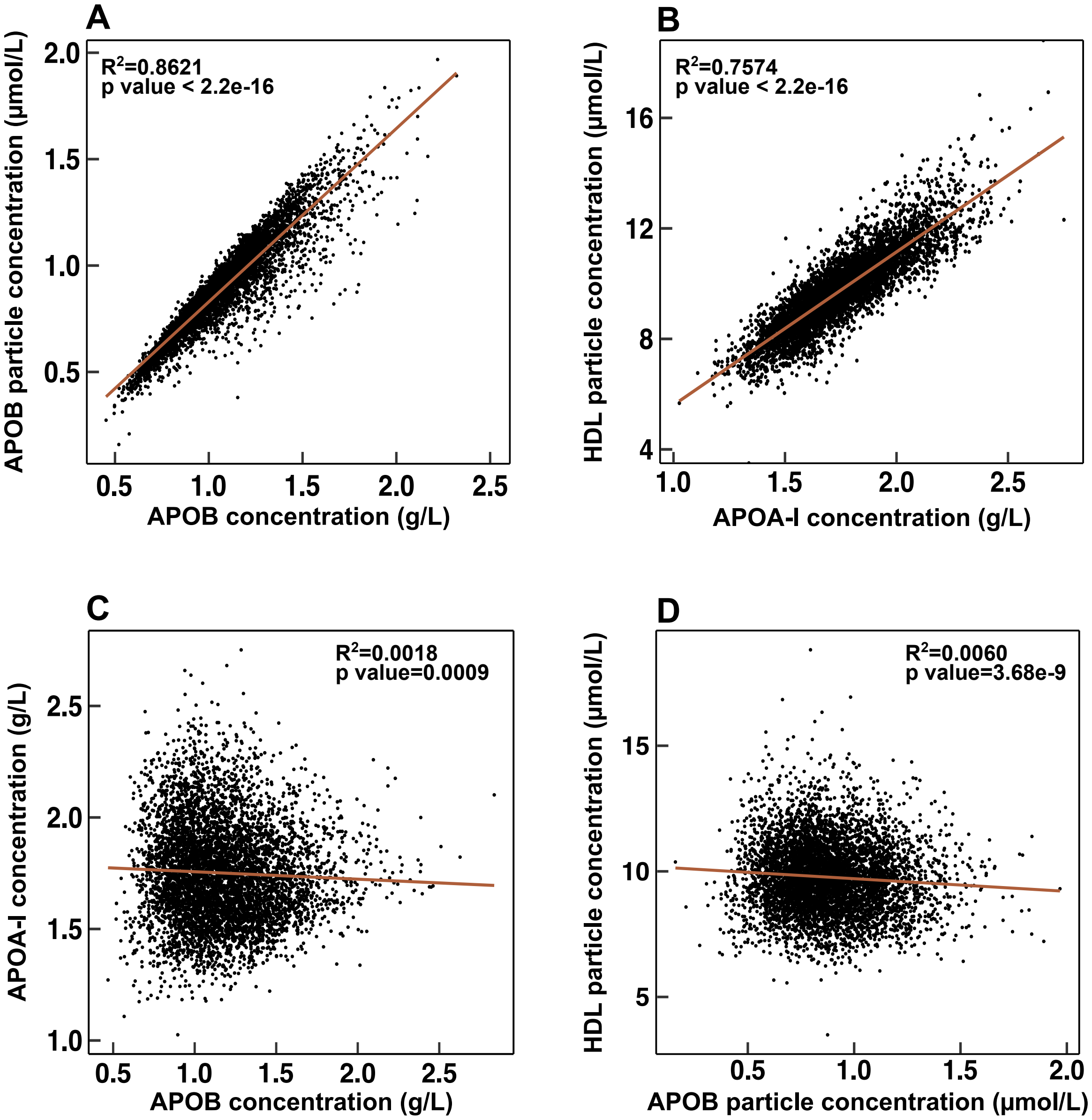

Figure S7

Scatter plots for the 5,651 participants in NFBC66: **A.** Apolipoprotein B concentration (g/L) vs apolipoprotein B particle concentration (µmol/L). **B.** Apolipoprotein A-I concentration (g/L) vs HDL particle concentration (µmol/L). **C.** Apolipoprotein B concentration (g/L) vs apolipoprotein A-I concentration (g/L). **D.** Apolipoprotein B particle concentration (µmol/L) vs HDL particle concentration (µmol/L).

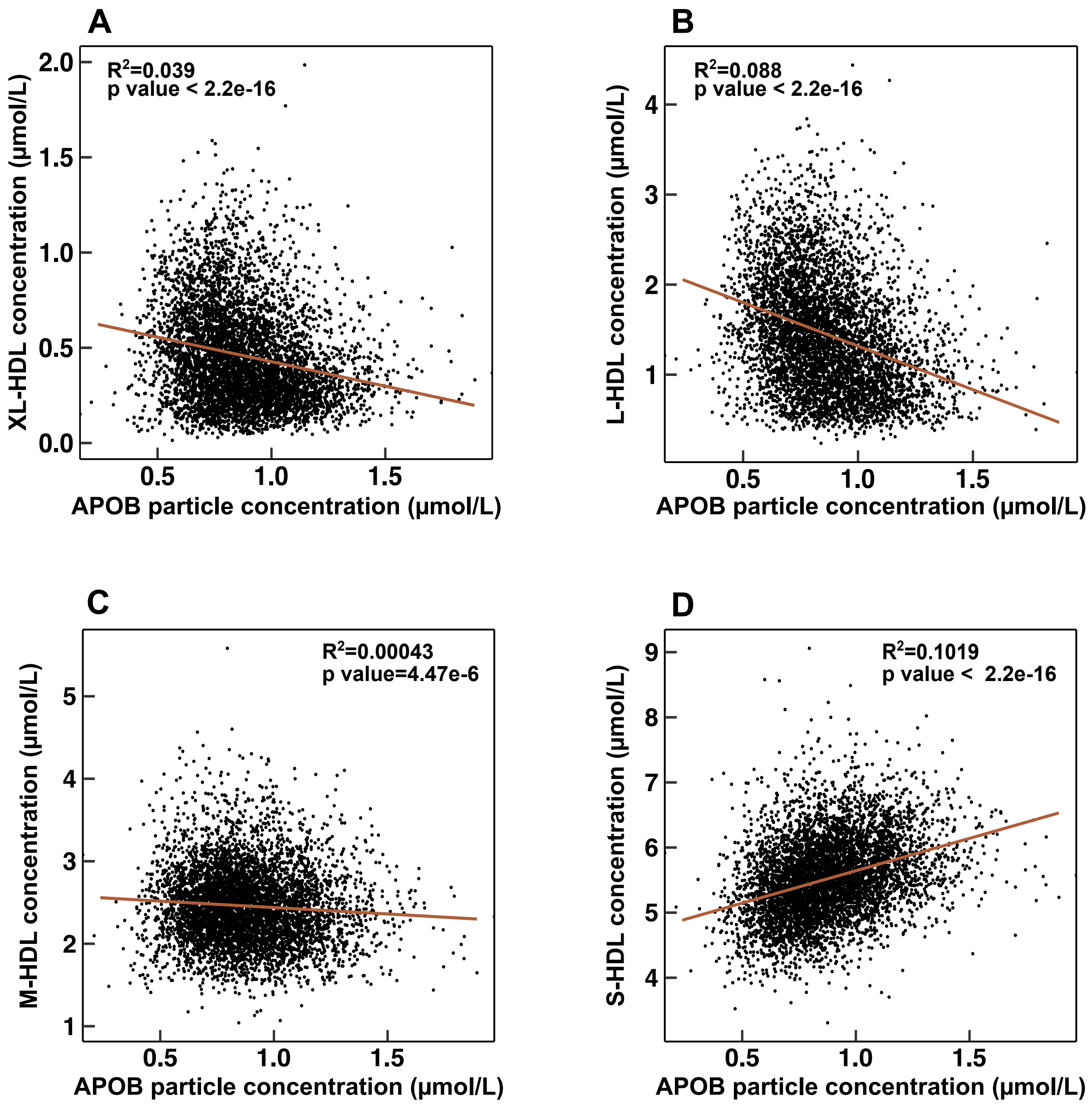

Figure S8.

Scatter plots for the 5,651 participants in NFBC66: **A.** Apolipoprotein B particle concentration vs XL-HDL particle concentration. **B.** Apolipoprotein B particle concentration vs L-HDL particle concentration. **C.** Apolipoprotein B particle concentration vs M-HDL particle concentration. **D.** Apolipoprotein B particle concentration vs S-HDL particle concentration.

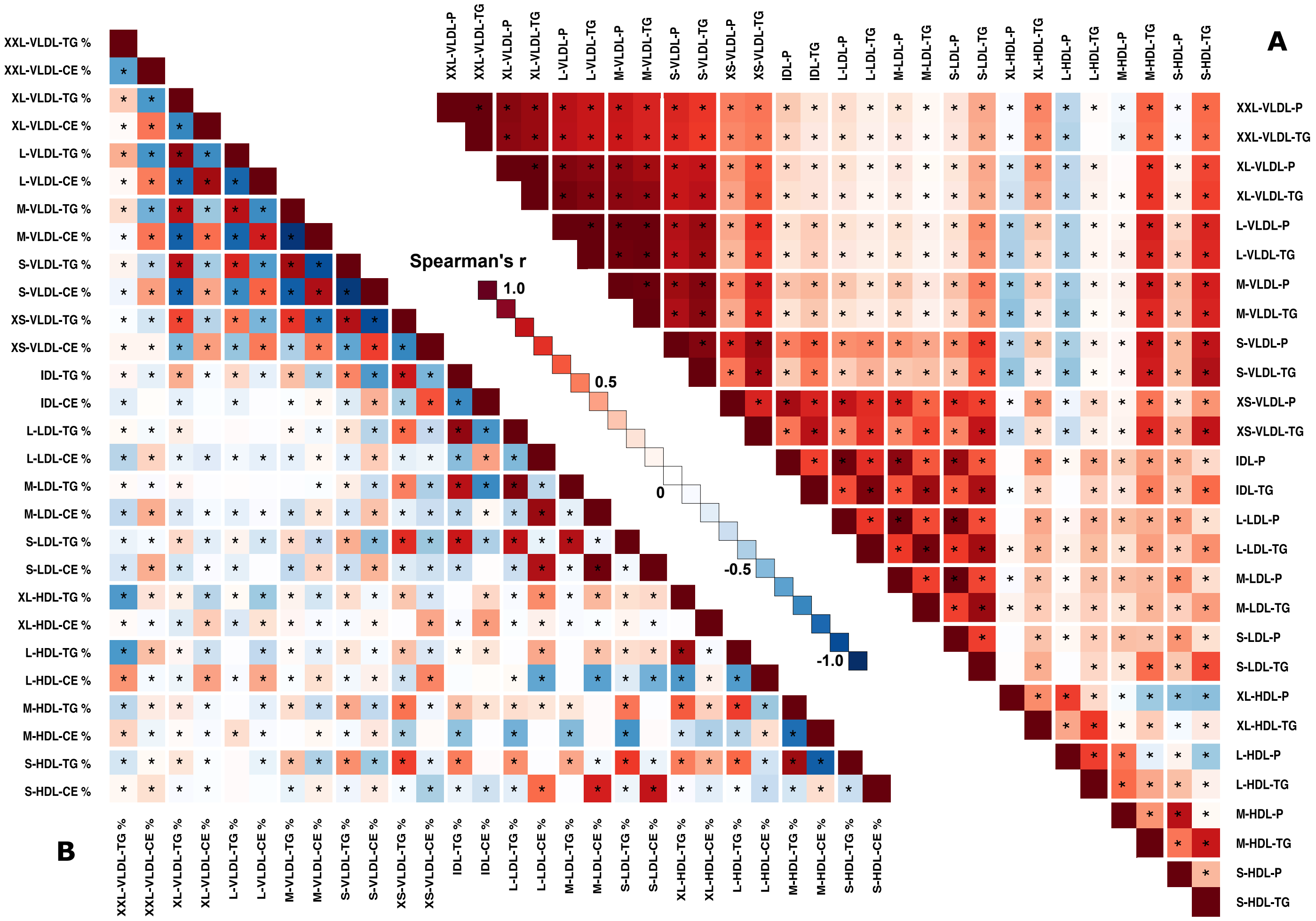

Figure S9

The associations between lipoprotein subclass particle and triglyceride concentrations (A) and between lipoprotein subclass lipid composition for triglycerides and cholesteryl esters (B). The data and abbreviations are as explained in the caption for Figure S6.

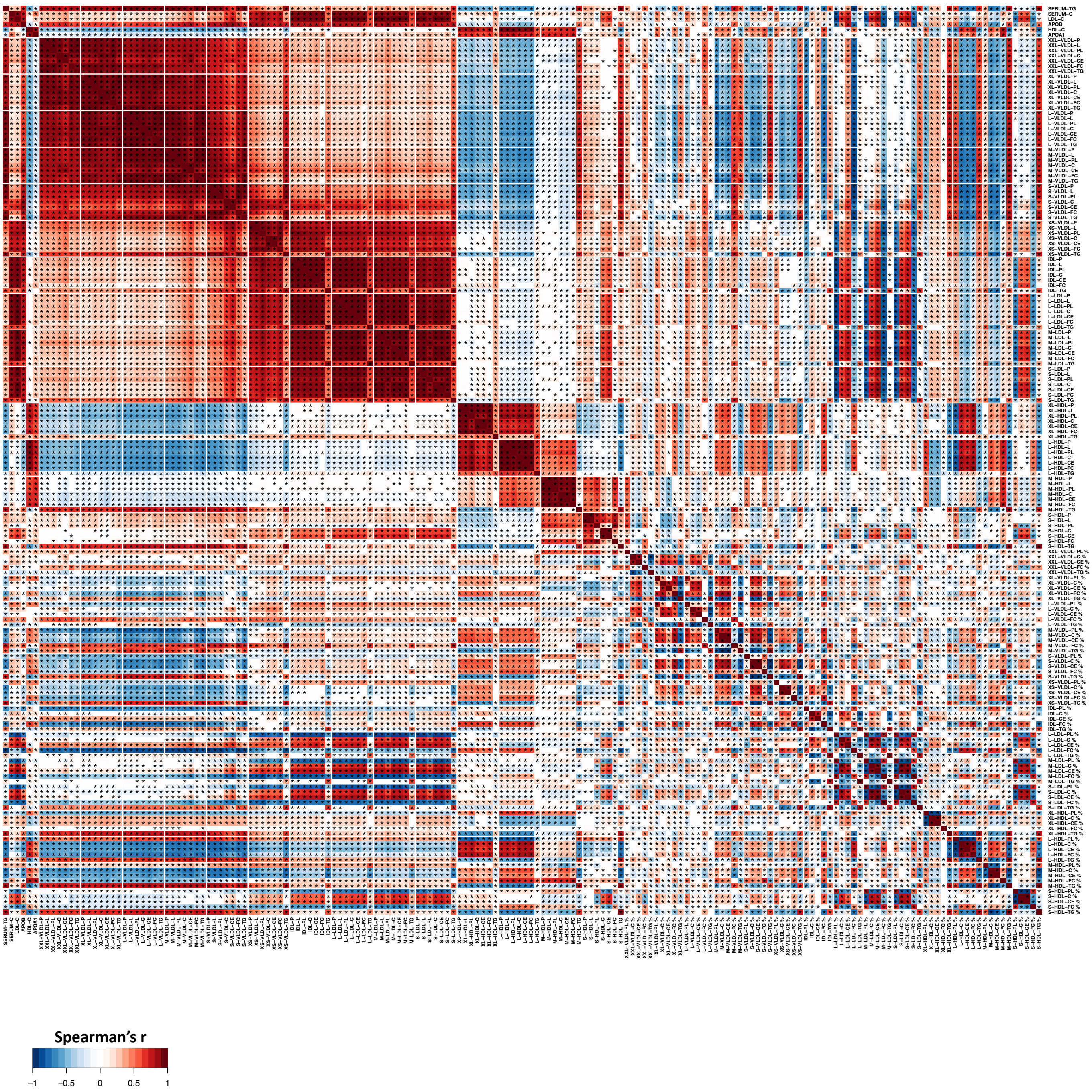

Figure S10.

A heatmap of Spearman's rank correlations (adjusted for sex) for the lipoprotein subclass concentration (98), composition (70) and standard lipid (4) measures as well as apolipoprotein B and A-I for the 5,651 participants in NFBC66 (Table S1). The abbreviations are as explained in the caption for Figure S6. L as part of the abbreviation for a subclass measure refers to total lipid concentration and P for total particle concentration.

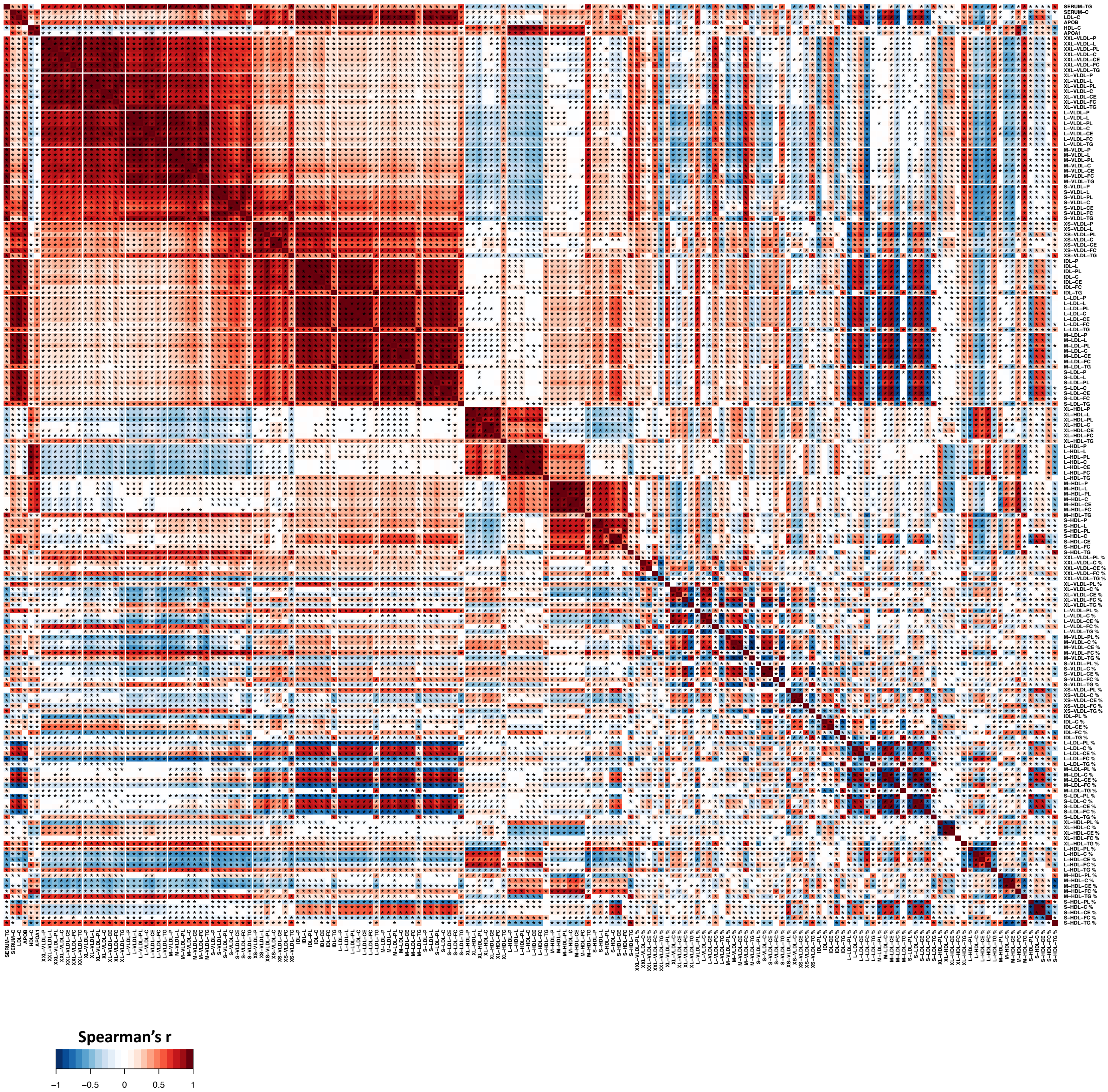

Figure S11. A heatmap of Spearman's rank correlations (adjusted for sex) for the lipoprotein subclass concentration (98), composition (70) and standard lipid (4) measures as well as apolipoprotein B and A-I for the 5,605 participants in NFBC86 (Table S2). The abbreviations are as explained in the caption for Figure S6. L as part of the abbreviation for a subclass measure refers to total lipid concentration and P for total particle concentration.

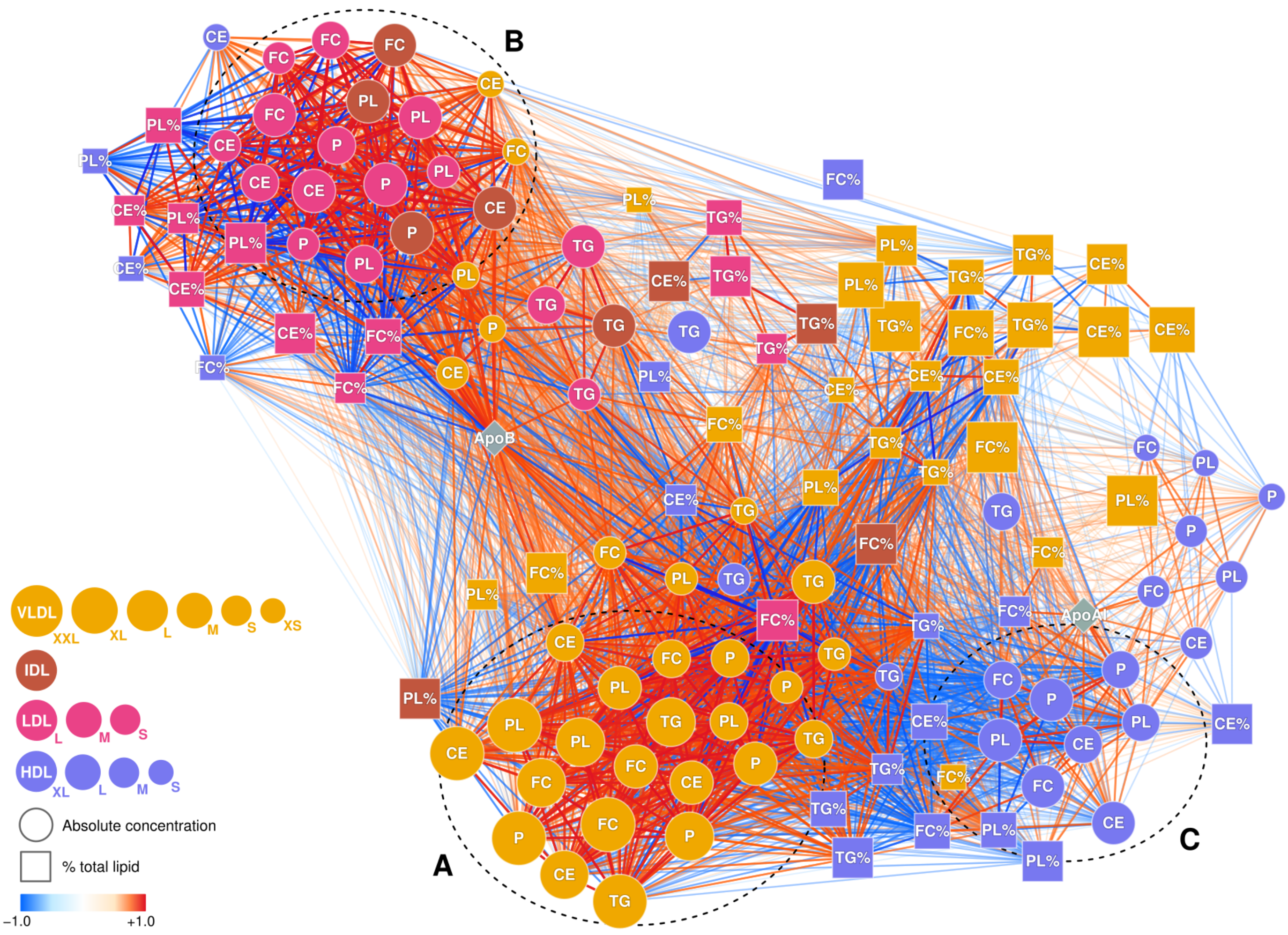

Figure S12. A Spearman's rank correlation network between lipoprotein subclasses for the 5,651 participants in NFBC66. All correlations with R^2 > 10% are shown in the plot. Any edges within a subclass were excluded. Derived measures including total lipid and total cholesterol were also excluded. The visualization was created by a modified version of the Fruchterman-Reingold force-directed node-positioning algorithm. Three densely connected groups of variables were observed: **A)** Lipid and particle concentrations of VLDL subclasses, **B)** Lipid and particle concentrations of IDL and LDL subclasses, **C)** Lipid and particle concentrations of the three largest HDL subclasses. The VLDL group (**A**) is positively connected to the IDL/LDL group (**B**) via the apolipoprotein B node in the middle, reflecting the apolipoprotein B particle cascade and metabolic continuum from the triglyceride-rich VLDL subclasses (**A**) to the cholesterol-rich IDL and LDL particles (**B**) in the circulation. The HDL subclass group (**C**) is inversely correlated with the VLDL subclass group (**A**) as an overall reflection of the

population level negative correlation between circulating triglycerides and HDL cholesterol. Apolipoprotein A-I node is widely connected among all the HDL subclasses but does not constitute a strong metabolic linkage as apolipoprotein B. The cholesterol and phospholipid compositions of the smallest HDL subclass are strongly connected to the IDL/LDL group (**B**) while the triglyceride composition and concentration are attached to both VLDL subclasses (**A**) and the other HDL subclasses (**C**). Triglyceride concentrations and subclass compositions are strongly positively linked throughout the entire network and among almost all the subclasses demonstrating wide-ranging spillover of circulating triglycerides.

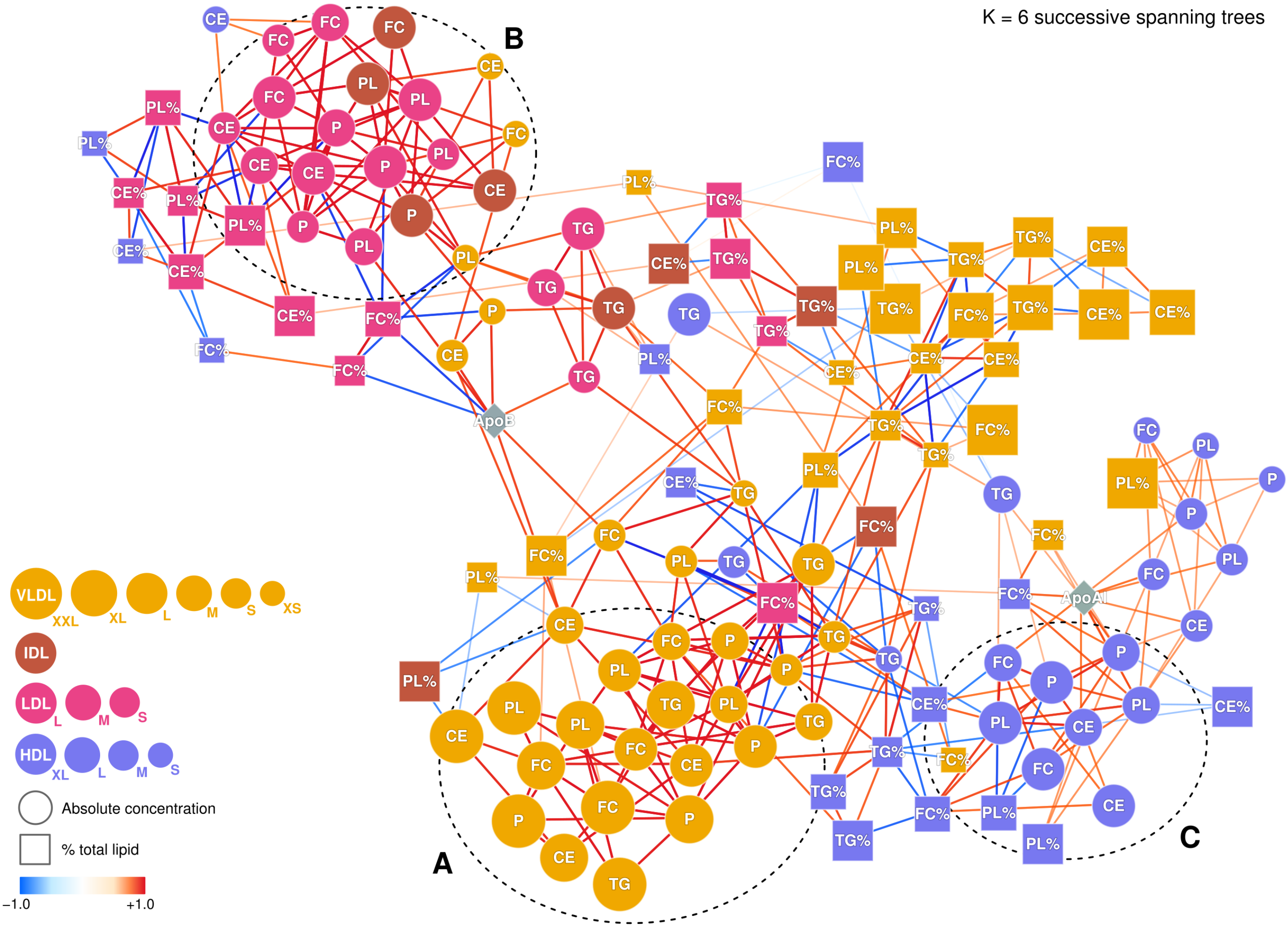

Figure S13. A Spearman's rank correlation network between lipoprotein subclasses for the 5,651 participants in NFBC66. A pruned version of the network show in Figure S12. Please see caption for Figure S12 for details. The pruned network was produced by a soft thresholding method introduced by Mäkinen et al.[3] The method works by removing the maximal spanning tree from the original network and using it as the start of a new network. Next, another max tree is removed from the remainder and added to the new network and so on. For the new figure here, this process was repeated six times. Consequently, the edges chosen for the figure depend on the connectivity of the network rather than the correlation coefficient of an individual edge. For example, if a node is connected to the rest of the network only via weak links, then the strongest of the weak links will be included in the pruned version even if R^2 would be below the hard threshold. Of note, the colour scale was chosen such that any $R^2 < 10\%$ will be effectively invisible and not drawn on the plot.

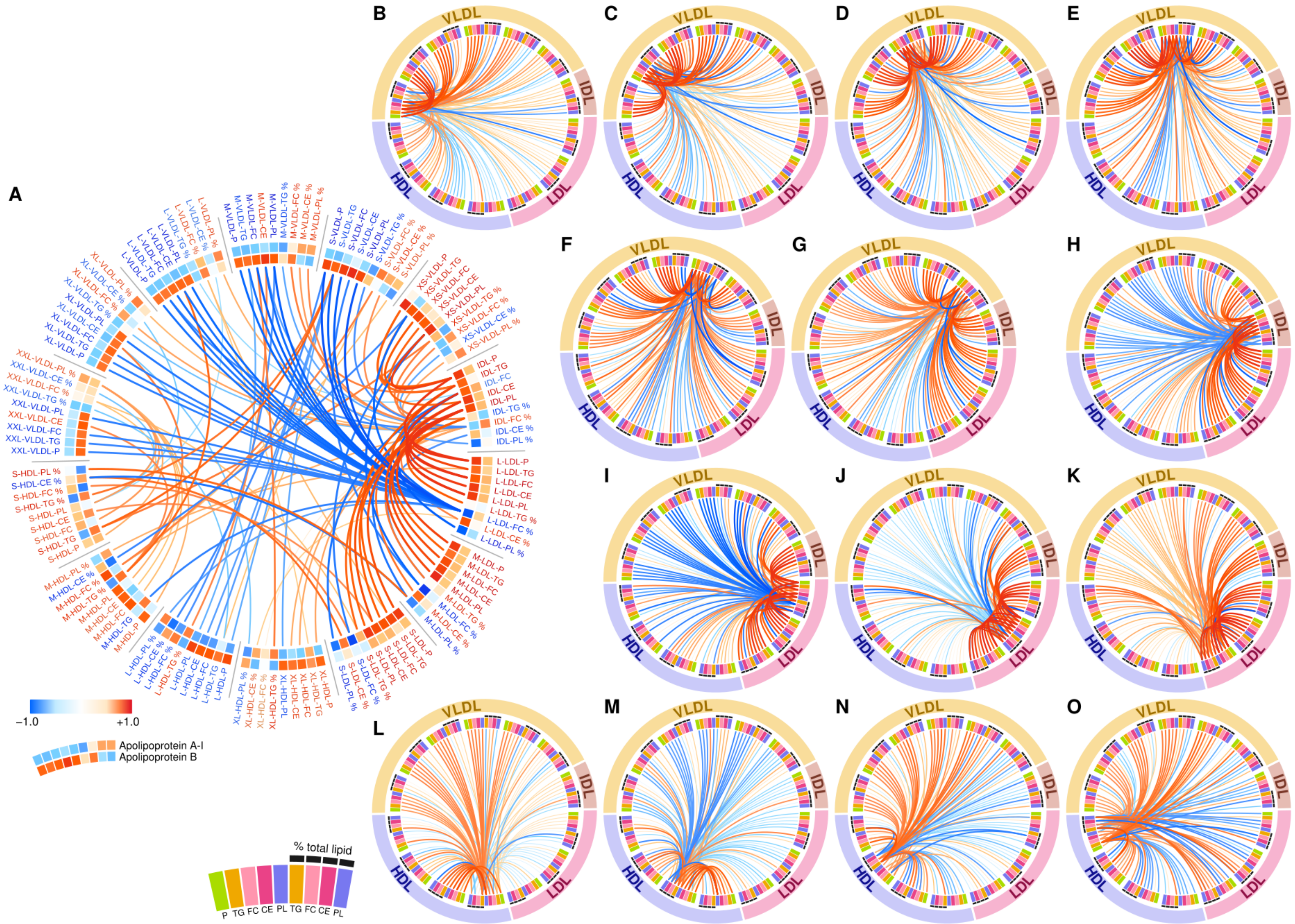
A
B
C
D
E
F
G
H
I
J
K
L
M
N
O
VLDL
IDL
LDL
HDL
XXL-VLDL-PL %
XXL-VLDL-CE %
XXL-VLDL-FC %
XXL-VLDL-TG %
XXL-VLDL-PL
XXL-VLDL-CE
XXL-VLDL-FC
XXL-VLDL-TG
XXL-VLDL-P
XL-VLDL-PL %
XL-VLDL-CE %
XL-VLDL-FC %
XL-VLDL-TG %
XL-VLDL-PL
XL-VLDL-CE
XL-VLDL-FC
XL-VLDL-TG
XL-VLDL-P
L-VLDL-PL %
L-VLDL-CE %
L-VLDL-FC %
L-VLDL-TG %
L-VLDL-PL
L-VLDL-CE
L-VLDL-FC
L-VLDL-TG
L-VLDL-P
M-VLDL-P
M-VLDL-TG
M-VLDL-FC
M-VLDL-CE
M-VLDL-PL
M-VLDL-TG %
M-VLDL-FC %
M-VLDL-CE %
M-VLDL-PL %
S-VLDL-P
S-VLDL-TG
S-VLDL-FC
S-VLDL-CE
S-VLDL-PL
S-VLDL-TG %
S-VLDL-FC %
S-VLDL-CE %
S-VLDL-PL %
XS-VLDL-P
XS-VLDL-TG
XS-VLDL-FC
XS-VLDL-CE
XS-VLDL-PL
XS-VLDL-TG %
XS-VLDL-FC %
XS-VLDL-CE %
XS-VLDL-PL %
IDL-P
IDL-TG
IDL-FC
IDL-CE
IDL-PL
IDL-TG %
IDL-FC %
IDL-CE %
IDL-PL %
L-LDL-P
L-LDL-TG
L-LDL-FC
L-LDL-CE
L-LDL-PL
L-LDL-TG %
L-LDL-FC %
L-LDL-CE %
L-LDL-PL %
M-LDL-P
M-LDL-TG
M-LDL-FC
M-LDL-CE
M-LDL-PL
M-LDL-TG %
M-LDL-FC %
M-LDL-CE %
M-LDL-PL %
S-LDL-P
S-LDL-TG
S-LDL-FC
S-LDL-CE
S-LDL-PL
S-LDL-TG %
S-LDL-FC %
S-LDL-CE %
S-LDL-PL %
XL-HDL-P
XL-HDL-TG
XL-HDL-FC
XL-HDL-CE
XL-HDL-PL
XL-HDL-TG %
XL-HDL-FC %
XL-HDL-CE %
XL-HDL-PL %
L-HDL-P
L-HDL-TG
L-HDL-FC
L-HDL-CE
L-HDL-PL
L-HDL-TG %
L-HDL-FC %
L-HDL-CE %
L-HDL-PL %
M-HDL-P
M-HDL-TG
M-HDL-FC
M-HDL-CE
M-HDL-PL
M-HDL-TG %
M-HDL-FC %
M-HDL-CE %
M-HDL-PL %
S-HDL-P
S-HDL-TG
S-HDL-FC
S-HDL-CE
S-HDL-PL
S-HDL-TG %
S-HDL-FC %
S-HDL-CE %
S-HDL-PL %
−1.0
+1.0
Apolipoprotein A-I
Apolipoprotein B
% total lipid
P TG FC CE PL TG FC CE PL

Figure S14. Circular visualisations of the strongest Spearman's rank correlations **A)** between lipoprotein subclasses and apolipoprotein A-I and B and **B–O)** between lipoprotein subclasses for the 5,651 participants in NFBC66. The colour scale and edge width indicate the value of the correlation coefficient. To reduce clutter in **A)** only edges between the major lipoprotein fractions VLDL, IDL, LDL and HDL are included (*i.e.*, correlations between lipoprotein subclasses with a major lipoprotein fraction are not shown) and only the maximal spanning tree by Spearman's R^2 of the remaining network is shown. In **B–O)** only edges connecting a specific lipoprotein subclass to other subclasses are included and the maximal R^2 spanning tree is depicted; the plots are drawn in the order of particle size from XXL-VLDL to S-HDL. The circular plot **A)** clearly depicts the strong positive associations between apolipoprotein B and all those lipoprotein subclass particle and lipid concentrations that contain apolipoprotein B (the inner circle). The strongest correlations for apolipoprotein A-I are with the concentration measures for XL-, L- and M-HDL. The overall negative correlation between circulating triglycerides and HDL cholesterol is reflected in the associations for both apolipoprotein B and apolipoprotein A-I. The circular plots **B–O)** elucidate the intricate change in the metabolic connections for different lipoprotein subclasses. A notable cut-off point in the apolipoprotein B-containing subclass particle metabolism is between XS-VLDL and IDL subclasses; IDL and L-LDL particles have a similar association pattern, but a rather different scheme takes place for S-LDL. Each of the HDL subclasses shows a rather tangled individually characteristic association pattern; strong positive correlations between S-HDL and various VLDL subclass concentrations are one notable feature.
